# Agentic LLM-based robotic systems for real-world applications: a review on their agenticness and ethics

**DOI:** 10.3389/frobt.2025.1605405

**Published:** 2025-08-19

**Authors:** Emmanuel K. Raptis, Athanasios Ch. Kapoutsis, Elias B. Kosmatopoulos

**Affiliations:** ^1^ Information Technologies Institute, The Centre for Research and Technology Hellas, Thessaloniki, Greece; ^2^ Department of Electrical and Computer Engineering, Democritus University of Thrace, Xanthi, Greece

**Keywords:** agentic AI, large language models (LLMs), autonomous robots, intelligent machines, ethical AI, AI transparency, human-robot interaction, real-world applications

## Abstract

Agentic AI refers to autonomous systems that can perceive their environment, make decisions, and take actions to achieve goals with minimal or no human intervention. Recent advances in Large Language Models (LLMs) have opened new pathways to imbue robots with such “agentic” behaviors by leveraging the LLMs’ vast knowledge and reasoning capabilities for planning and control. This survey provides the first comprehensive exploration of LLM-based robotic systems integration into agentic behaviors that have been validated in real-world applications. We systematically categorized these systems across navigation, manipulation, multi-agent, and general-purpose multi-task robots, reflecting the range of applications explored. We introduce a novel, first-of-its-kind agenticness classification that evaluates existing LLM-driven robotic works based on their degree of autonomy, goal-directed behavior, adaptability, and decision-making. Additionally, central to our contribution is an evaluation framework explicitly addressing ethical, safety, and transparency principles—including bias mitigation, fairness, robustness, safety guardrails, human oversight, explainability, auditability, and regulatory compliance. By jointly mapping the landscape of agentic capabilities and ethical safeguards, we uncover key gaps, tensions, and design trade-offs in current approaches. We believe that this work serves as both a diagnostic and a call to action: as LLM-empowered robots grow more capable, ensuring they remain comprehensible, controllable, and aligned with societal norms is not optional—it is essential.

## 1 Introduction

*“I propose to consider the question, ‘Can machines think?”’*, this is how Alan Turing, in 1950, began his first published paper focusing exclusively on machine intelligence. Rather than trying to determine if a machine is thinking, Turing proposed the well-known Imitation Game, which led to the foundation for Natural Language Processing (NLP) systems designed to imitate human conversation.

Modern Large Language Models (LLMs) have opened new avenues for enhancing robot intelligence and autonomy by enabling more natural human-robot interactions Zeng et al. (2023). Unlike traditional robotic systems with hand-coded dialogue or fixed responses, LLMs can understand and generate open-ended natural language, allowing robots to engage in human-like conversation and complex instruction following. This embodiment of LLMs means using them as part of a robot’s control loop or “brain,” so the robot benefits from the vast knowledge and reasoning capabilities learned from text. Researchers have recognized the promise of this approach in improving robots’ decision-making, planning, and adaptability.

However, bridging purely text-based LLMs with physically embodied robots poses significant challenges. A major limitation is that most LLMs rely on textual input/outputs, which is insufficient for robots that must perceive images, navigate spaces, and manipulate objects. While LLMs are designed to understand, generate, and process human language, they often lack true comprehension of commonsense or real-world knowledge, leading to potentially illogical or even biased outputs based on their training data, escalating the ongoing debate on whether they actually “think” or merely generate statistical predictions based on patterns in data. This highlights one of the biggest challenges in robotics today—developing systems that not only process and generate language but also achieve true intelligence by grounding their understanding in real-world perception, enabling them to sense, interpret, and make decisions in complex, real-world environments.

To overcome these challenges, agentic AI, the next frontier in artificial intelligence, is poised to bridge the gap between passive computation and true autonomy. Unlike conventional AI models that passively generate responses from learned patterns, “agentic AI” refers to an artificial intelligence system that can act as an autonomous agent with the capacity to perceive its environment, make decisions, and perform actions to achieve goals with minimal or no human intervention. In robotics, this concept signals a shift from machines that merely execute pre-programmed commands toward autonomous agents with higher-level cognitive capabilities. This introduction provides context for our survey by defining agentic AI and highlighting why integrating LLMs into robotic systems could accelerate this transformation.

**FIGURE 1:**
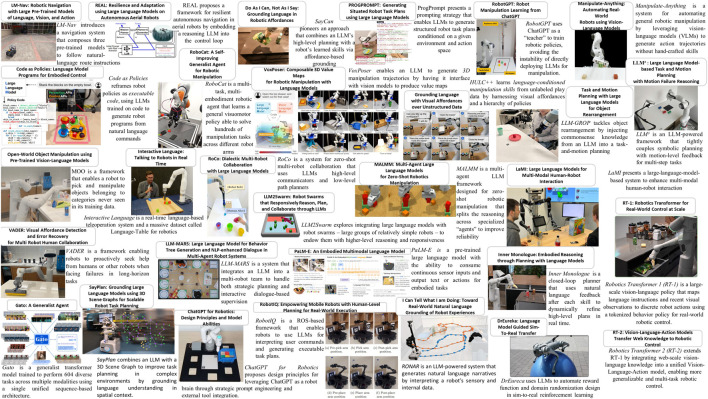
A visual summary of recent Agentic LLM-based robotic systems reviewed in this survey.

Recent work addresses this by feeding multimodal inputs to LLMs or by coupling LLMs with perception modules (see Figure 1). For example, PaLM-E (Driess et al., 2023) is a 562-billion parameter embodied multimodal model that accepts continuous visual observations alongside text, effectively bridging the gap between language and perception. By treating images and other sensor readings as just another language” input, PaLM-E enables an LLM to reason about the physical environment and output action instructions. This represents a step toward true embodiment, as the LLM’s internal knowledge is grounded in real-world context.

Several research groups have also proposed system architectures to integrate agentic LLMs into robots. A common pattern is to use the LLM for high-level reasoning and task decomposition while traditional robotics modules handle low-level control. In Google’s PaLM-SayCan system (Ahn et al., 2022), a pre-trained LLM (PaLM) is coupled with a set of pre-learned robotic skills and an affordance function. The LLM provides semantic understanding of an open-ended instruction, breaking it into feasible steps, and the affordance model estimates which actions are physically possible in the current context. This way, the robot “acts as the language model’s hands and eyes” while the LLM supplies high-level reasoning. Beyond specific implementations, conceptual frameworks have been proposed for LLM-based robotic systems. Some works (e.g., RobotGPT framework) envision a robot “brain” where an LLM like ChatGPT orchestrates perception and control modules for multi-modal understanding and action (Jin et al., 2024). Social robotics researchers foresee replacing traditional pipelines (speech recognition → intent parsing → dialog management) with unified LLM-based architectures for more fluid interactions. Despite these visions, embodiment remains an open challenge: an effective integration requires tight coupling between an LLM’s abstract reasoning and the robot’s continuous sensorimotor experience. Ensuring real-time responsiveness, grounding in perceptual reality, and handling the physical constraints of robots are ongoing research problems.

### 1.1 Contributions

Prior surveys often catalog impressive robotic behaviors that unfortunately only exist in simulation; here we aim to highlight which approaches have actually been deployed on physical robots and what outcomes were observed. More specifically, this survey paper aims to fill the gaps identified above and go beyond the current literature, by providing an extensive collection of agentic LLM-based robotic systems and set its boundaries for easier understanding by a wider audience of researchers, developers, and policymakers.

We identified relevant papers by searching scholarly databases (e.g., Scopus, Web of Science, IEEE Xplore, ACM Digital Library, arXiv) using keywords like “LLMs in robotics”, “Agentic LMMs”, “LLMs for human-robot interaction,” etc., focusing on the years 2022–2025. We included only those works where the LLM-enhanced robotic system was validated in real-world settings (as opposed to simulation-only studies) to ensure our review emphasizes practical deployments. From an initial list of candidates, we applied further inclusion criteria: works had to explicitly incorporate a large language model in the robot’s decision-making or control loop and exhibit at least one of the agentic characteristics (autonomy, goal-directedness, adaptability, decision-making). This process yielded 30 key papers that span diverse application domains. We acknowledge that while we aimed for comprehensive coverage, some relevant works might have been excluded; our selection prioritizes depth of analysis in real-world contexts. We provide the full list of reviewed papers both in Figure 1 and in Tables 2–5.

Key contributions of this survey include:

• A transparent survey methodology, resulting in a curated dataset of 30 recent papers at the intersection of LLMs and robotics, all validated in real-world contexts,

• A comprehensive study of the techniques and concepts used in the reviewed LLM-based robotic systems based on their task domain,

• A comparative agenticness classification of how each work embodies agentic” characteristics,

• An evaluation considering the ethical, societal, and regulatory issues with adopting agentic AI, including relevant concerns of responsibility, equity, and transparency,

• Recommendations towards future research, advancing suggestions on how the issues of scale, context, and ethics are best integrated into the implementation of agentic AI for real-world.

This paper’s expected contributions are more than simply a literature review; rather, they should offer a useful and well-organized background to classify the LLM-based robotic systems based on their agentic behavior and understand the ethical and transparency issues of agentic AI. This paper aims *to go beyond theory and address the real-world challenges of agentic AI in robotics to promote and sustain the development of ethical agentic AI systems.*

## 2 Related work: surveys on LLM integration in robotics

Recent survey papers reveal several overarching themes in integrating large language models (LLMs) into robotics and agentic systems (Zeng et al., 2023; Hu et al., 2023; Shavit et al., 2023; Kim Y. et al., 2024; Huang et al., 2024; Jeong et al., 2024; Bousetouane, 2025; Acharya et al., 2025; Li et al., 2025). Table 1 compares key themes across representative surveys, highlighting their focus areas and omissions. We discuss these surveys in terms of their scope, real-robot deployment, agentic AI aspects, and treatment of ethics.

**TABLE 1:** Comparison of existing surveys on LLM-based robotics, underscoring their coverage of real-world applications, agentic autonomy, and ethical considerations.

Survey	Scope and focus	Real-robot deployment	Agentic AI	Ethics and transparency
Zeng et al. (2023)	Broad overview of LLM applications in robotics; positions LLMs as enhancing “embodied intelligence”	Reviews research prototypes by acknowledging sim-to-real gap; no in-depth deployment evaluation	Agents as embodied AI; does not analyze adaptive autonomy in open-ended environments	Briefly notes societal implications; little on bias or accountability; ethical discussion is philosophical and short-term safety is only mentioned in passing
Hu et al. (2023)	Survey and meta-analysis of foundation models (NLP/CV) in robotics; proposes taxonomy and aggregates experimental results	Motivated by sim-to-real gaps; summarizes research outcomes rather than long-term deployment	Envisions “general-purpose” robotic agents; it stops short of examining cognitive autonomy or continuous learning in depth	Focuses on performance metrics; ethical implications, bias or safety receive minimal attention; Governance and transparency considerations are out of scope
Shavit et al. (2023)	Defines agentic AI systems; provides safety best practices and governance frameworks for ensuring responsible deployment	Focuses on outlining governance frameworks rather than empirical deployment evaluations	Clarifies what constitutes agentic AI from a governance perspective; no technical adaptive autonomy	Strong focus on safety, accountability, and transparency; oriented toward policy recommendations
Kim et al. (2024b)	Component-wise integration of LLMs into robotics; practical guidelines for prompt engineering and system design	Focuses on methodology; no empirical deployment studies included	Covers autonomy via planning/control components; agentic behavior is not a dedicated topic	Notes the need for output filtering; no in-depth coverage of ethics, bias, transparency or regulatory issues
Huang et al. (2024)	Cross-domain review (robotics, healthcare, gaming) for Agent AI”; analyzes architectures for comprehensive intelligence	Offers conceptual frameworks with theoretical scenarios; lacks real-world deployment data	Emphasizes on holistic and adaptive intelligence; fully agentic systems that learn and evolve continuously	Addresses ethical challenges and the need for transparency; discusses accountability and oversight
Jeong et al. (2024)	Review of how foundation models (LLMs and VLMs) enhance robot intelligence; emphasizes LLMs’ generalization in real-world	Relates to physical-world scenarios; evaluation of long-term robustness or field trials is not systematically presented	Discusses embodied intelligence; not explicitly framed as agentic.”	Strong focus on safety and ethics: warns of biased or unsafe outputs from LLM-powered robots and the risk of misuse (violent/illegal instructions)
Bousetouane (2025)	Industrial applications of vertical AI agents; emphasizes practical design and deployment strategies	Strong emphasis on integration and real-world challenges	Targets agentic system design and adaptive decision-making for real-world	Industrial applicability over an in-depth exploration of ethical issues and transparency measures
Acharya et al. (2025)	Reviews architectures and methods for autonomous intelligence and agentic AI in robotics	Mostly conceptual architectural views; minimal real-world deployment emphasis	Addresses agentic behavior via goal-directed behavior and decision-making	Covers safety and accountability at a high level; lacks detailed discussion on transparency and ethical governance
Li et al. (2025)	Focuses on multi-robot coordination via LLMs	Identifies adaptability and latency as real-world challenges; forward-looking rather than reporting real-world deployments	Covers collaborative and adaptive behaviors; lack of learning strategies autonomously over time	Notes safety features in LLMs; does not delve into multi-robot ethical governance

### 2.1 Scope of existing surveys

Each survey has a distinct scope, and collectively, they reveal important gaps. Some surveys provide broad taxonomies of applications–for instance, Zeng et al. (2023) lays the foundation with a broad taxonomy of core robotics functions—such as control, perception, planning, and navigation—framing LLMs as enhancers of embodied intelligence. Others focus on specific aspects: Hu et al. (2023) narrows the focus by employing a meta-analytical approach to assess foundation models in NLP and computer vision, particularly emphasizing experimental outcomes and sim-to-real challenges in general-purpose robot skills. Shavit et al. (2023) defines agentic AI systems and outlines safety and accountability best practices, with less emphasis on technical adaptive autonomy, though. Kim Y. et al. (2024) take a methodology-centric view, breaking down robotics into components (communication, perception, planning, control) and offering integration guidelines for prompt engineering to enable newcomers to access LLM-based robotics solutions. Huang et al. (2024) spans multiple domains, including robotics, healthcare, and gaming, to propose holistic architectures for continuously evolving agents. Jeong et al. (2024) emphasizes how foundation models (including both LLMs and vision-language models-VLMs) improve various robotics subdomains like reward design in reinforcement learning, low-level control, high-level planning, manipulation, and scene understanding. Bousetouane (2025) provides a comprehensive introduction to agentic systems and actionable insights for deploying vertical AI agents in driving industry-specific applications, while Acharya et al. (2025) explores the foundational concepts, unique characteristics, and core methodologies of agentic AI across various fields, including healthcare, finance, and adaptive software systems, emphasizing the advantages of deploying agentic systems in real-world scenarios, outlining also the ethical challenges related to goal alignment, resource constraints, and environmental adaptability. Last but not least, Li et al. (2025) rounds out the collection by examining multi-robot coordination via LLMs, identifying challenges like scalability and latency primarily through simulation-based studies.

### 2.2 Real-robot deployment

Another shortcoming of prior surveys is their limited treatment of LLM-based robotic systems in real-world deployment. Most of the surveys discuss systems that have only been tested in simulation or controlled lab settings. For example, Zeng et al. (2023) acknowledge that training robotics models purely in games or simulators often fails to translate to real environments–a model with 90% success in simulation might drop to 10% in reality. They cite this sim-to-real gap as a challenge, noting issues like the cost of real-world data collection and the poor transferability of policies trained in virtual settings. However, while they raise the point, the survey does not provide a systematic review of how current research has tried to bridge this gap while remaining an identified problem rather than an analyzed one. Many surveys share this pattern: real-world applicability is acknowledged as a challenge, but not rigorously evaluated. On the other hand, across Table 1, prior surveys lean towards describing architectures and potential applications, with relatively few references to outcomes of real-world experiments. For instance, Jeong et al. (2024) include scattered examples of robotics experiments (such as a mobile manipulator executing plans from natural language, or Google’s RT-2 model for vision-language-action). Yet, the survey still does not compile results from those physical deployments into an analysis of how well current LLM-based robots actually perform when faced with the messiness of reality. Concisely, the prior surveys provide a strong foundation of concepts and initial demonstrations of LLM-based robotic systems, but a survey with a decidedly real-world, deployment-oriented viewpoint is needed to push the field from promising research to impactful practice; which approaches have actually been tested in real robots, what are the outcomes in terms of their agenticness, and what practical recommendations can be made. This new perspective would complement the existing literature by focusing on real-world applicability of LLM-based robotic systems–the very aspect that prior surveys largely left as an open challenge.

### 2.3 Agentic AI overlooked

Despite this coverage, critical aspects are overlooked. The idea of *agentic AI*–robots with autonomy, goal-directed behavior, adaptability, and decision-making – is only superficially addressed in existing surveys. Most prior works frame LLM-equipped robots as improved versions of traditional robots, not as fundamentally new agents with higher-level cognitive autonomy. Most of the surveys, for instance Kim Y. et al. (2024), structure their survey around integrating LLMs into four robotics components: communication (language understanding/generation), perception, planning, and control. This provides a valuable breakdown of where LLMs can slot into robot architectures, and the authors offer practical prompt-engineering tips for each component. Yet, this component-wise approach means the survey stops short of examining whole-agent behavior that emerges when these pieces work together. In other words, the surveys tend to discuss LLM-based robotic systems in constrained task contexts (e.g., following instructions, generating plans) rather than as continually learning agents operating in open-ended environments. There is little discussion of robots exhibiting long-horizon autonomy, goal-directed behavior, adaptability, and decision-making–hallmarks of “agentic” AI. In summary, each existing survey provides pieces of the puzzle (task planning, language-based control, human-robot interaction improvements, etc.), but none squarely focus on the adaptive agency aspect. This is a notable gap: as the community moves toward more autonomous robot agents, guidance on how LLMs contribute to capabilities like autonomy, goal-directed behavior, adaptability, and decision-making is lacking in the survey literature.

### 2.4 Ethical, safety, and transparency considerations

Despite rapid advances in LLM-based robotic systems, existing surveys tend to address ethical, safety, and transparency concerns only in a cursory manner—leaving a significant gap in both theory and practice. As our analysis in Section 4 shows, most prior works mention these issues superficially without integrating detailed evaluation metrics such as fairness/bias, safety/robustness, transparency/interpretability, and governance/compliance.

For example, Kim S. S. et al. (2024) briefly notes the need for filtering and correction mechanisms to mitigate inaccurate or unexpected outputs, yet it does not elaborate on how such measures could be systematically evaluated or enforced. Similarly, Zeng et al. (2023) warns that biased or misconceived outputs from LLMs might lead to harmful physical actions—like a kitchen robot inadvertently causing a fire—or even raise data privacy issues when sensitive information is sent to the cloud. However, their discussion remains largely at the level of risk identification rather than proposing concrete mitigation strategies or accountability frameworks. In contrast, Jeong et al. (2024) offers a more direct treatment by detailing how LLMs can embed biases (e.g., biases related to race and gender) and may output unsafe instructions. Even so, as highlighted in our ethical evaluation framework (Sections Section 4.2; Section 4.3), this survey stops short of exploring how to audit an LLM-driven robot for bias or how to ensure accountability when decision-making is partially autonomous. Notably, none of the reviewed surveys propose robust ethical governance measures—such as explainability modules, comprehensive audit logs, or human-in-the-loop oversight—that are essential for achieving traceability and accountability in real-world applications.

Moreover, transparency—a critical factor for building user trust in autonomous systems—is rarely discussed beyond superficial mentions. Few surveys analyze whether users can effectively interrogate the robot’s reasoning process or if the robot can provide intelligible explanations for its actions. This is particularly concerning given that, as LLM-based robots transition from controlled lab environments to dynamic public settings, the practical implications of bias, accountability, and transparency become immediate and multifaceted. Emerging regulatory frameworks (e.g., the EU’s proposed AI Act) further underscore the need for stringent oversight and explainability in high-risk AI systems. Yet, to date, no survey fully connects these regulatory demands with current LLM-based robotics practices. As our meta-analysis reveals, existing literature often leaves readers with the simplistic takeaway of be careful, there are issues” without offering guidance on how to mitigate them.

In summary, the current surveys each cover pieces of the LLM-for-robotics puzzle, but none provides a comprehensive picture of LLM-based agentic AI in robotics. Critical aspects such as real-robot deployment, agentic AI capabilities, and depth of ethical, safety, and transparency considerations are not sufficiently discussed as standalone themes. This gap is significant because these aspects will determine whether LLM-based robotic systems can move beyond demos to become reliable, autonomous agents in the real world.

### 2.5 Preliminaries and definitions

In this survey, we review only agentic LLM-based robotics systems that have been validated in real-world applications. We define the agentic behavior of a robotic system based on the four following characteristics: i) Autonomy: The ability to operate without constant human intervention; ii) Goal-directed behavior: A focus on achieving specific outcomes based on a set of objectives; iii) Adaptability: The capacity to learn and adjust to new circumstances or information; iv) Decision-making capabilities: The ability to evaluate options and choose the best course of action based on available data. In this paper, we use the term agenticness to refer to the degree to which these characteristics are embodied in an LLM-based robotic system. We acknowledge that our agenticness ratings are based on the authors’ interpretations of each work; developing quantitative metrics for each characteristic would be an excellent direction for future research.

### 2.6 Paper outline

The remainder of this paper is organized as follows: Section 3 presents a thorough literature overview of the relatively recent LLM works and their integration into robotics. It also introduces a novel, first-of-its-kind agenticness classification across existing works regarding the notion of their agentic behavior, Section 4 outlines an evaluation framework for categorizing current LLM-based robotic systems in terms of ethics, safety, and transparency, covering aspects such as bias and fairness, robustness and safety mechanisms, human oversight, explainability, auditability, and regulatory compliance, Section 5 classifies the recent agentic LLM-based robotic systems according to both their level of agenticness and their alignment with ethical, safety, and transparency principles, and Section 6 provides an overview of the research and delves into its results and consequences.

## 3 LLM-based systems applied in real-world robotics

Recent advances in LLMs have opened new pathways to imbue robots with such agentic” behaviors, by leveraging the LLMs’ vast knowledge and reasoning capabilities for planning and control. More specifically, this survey provides a comprehensive review of 30 recent papers at the intersection of agentic AI and robotics for real-world. We organize the discussion into four task domains – (i) Navigation and Mobility, (ii) Manipulation and Object Interaction, (iii) Multi-Agent and Collaborative Robotics, and (iv) General-Purpose Multi-Task Robots–reflecting the range of applications explored. Each paper is assigned to its primary domain (and secondary domains, if applicable). In each subsection, we first overview the domain’s significance and the role of LLM-based agency, then summarize the key papers (detailing their methodology, results, and contributions), introducing terminologies and techniques for subsequent reviews. Finally, for each task domain, we introduce an agenticness classification framework to evaluate and compare how agentic these systems are based on four key characteristics: Autonomy, Goal-Directed Behavior, Adaptability, and Decision-Making. For each characteristic, we assigned a qualitative rating (Low 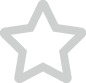, Moderate 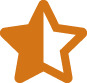, and High 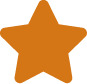) on each agentic dimension based on evidence in the papers. For example, a system that operates for prolonged periods without human intervention and can generate its own sub-goals would rate High in autonomy and goal-directed behavior. One that only executes pre-specified waypoints with continuous operator oversight would rate Low in Autonomy. Thus, we consider overall agenticness as a numeric average; we consider a system ‘High’ overall if it excels in most of the four characteristics without serious weakness in any. We present our comparative agenticness classification in the following Tables 2–5, grouped by domain.

**TABLE 2:** Agenticness classification for navigation and robot mobility.

Paper – Overall agenticness	Autonomy	Goal-directed behavior	Adaptability	Decision-making
Shah et al. (2023) (LM-Nav) 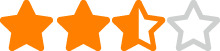	High – Navigates autonomously using LLM guidance once given an instruction	High – Follows long-horizon routes via intermediate landmarks	Moderate – Demonstrates zero-shot navigation in similar outdoor environments, limited by the pre-trained domain	Low – Combines LLM-generated sub-goals with a low-level navigation policy for sequential decisions
Tagliabue et al. (2024) (REAL) 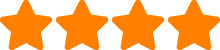	High – LLM adjusts controller parameters on the fly	High – Maintains mission goals and dynamically reconfigures to stay on task	High – Actively tunes flight parameters and re-plans in real time	High – Makes control decisions (like emergency landing) beyond the original design

**TABLE 3:** Agenticness classification for manipulation and object interaction.

Paper – Overall agenticness	Autonomy	Goal-directed behavior	Adaptability	Decision-making
Ahn et al. (2022) (SayCan) 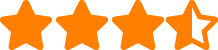	High – Autonomous high-level planning and execution via pre-defined skills	High – Decomposes user goals into feasible steps guided by an affordance model	Moderate – Limited to the provided skill set	High – Uses the LLM to plan step-by-step actions with a value function to choose the best action
Singh et al. (2023) (ProgPrompt) 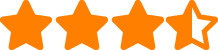	High – LLM generates plan-program and robot executes it without intervention	High – Focuses on completing the task defined by the prompt, producing a structured plan to reach the goal	Moderate – Generalizes planning logic to new object configurations if described; limited by planning knowledge in prompt (no learning new domain dynamics)	High – LLM decision-making in structured format: chooses sequence of action by reasoning over provided domain state
Jin et al. (2024) (RobotGPT) 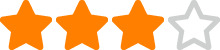	Moderate – Runtime policy is fixed; LLM used offline as teacher for stable execution	High – Trained policy reliably pursues user-specified tasks	Moderate – Policy inherits some generality but must be retrained for new tasks	High – LLM code guides the policy’s structure, leading to effective step-by-step manipulations
Duan et al. (2024) (Manipulate-Anything) 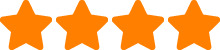	High – Autonomously plans multi-step manipulations with self-verification and retries	High – Breaks user instructions into sub-tasks, completes them sequentially	High – Handles diverse objects and recovers from failures via reattempts	High – Combines vision-language reasoning with motion planning to decide how, where, and when to act
Liang et al. (2023) (Code as Policies) 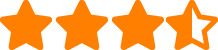	High – Autonomously generates and executes robotic control policies based on user input; constrained by the programming environment	High – Explicitly task-driven, focusing on achieving manipulation tasks	Moderate – Generalizes well across tasks but lacks real-time adaptability	High – Translates high-level commands into structured, executable policies with strong decision-making
Bousmalis et al. (2023) (RoboCat) 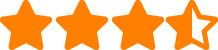	High – Executes manipulation tasks autonomously and self-improves via iterative retraining	High – Consistently pursues designated manipulation goals and a meta-goal of self-improvement	High – Rapidly adapts to new tasks and robot embodiments with minimal additional data	Moderate – Uses a decision transformer for sequential actions with meta-decision aspects in self-improvement
Huang et al. (2023) (VoxPoser) 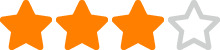	High – Generates continuous action maps autonomously using a combined LLM and vision-language model	High – Directly produces spatial action targets for manipulation goals	Moderate – Capable of zero-shot trajectory generation for novel spatial preferences within a limited set	Moderate – Computes value maps to determine precise action locations
Mees et al. (2023) (HULC++) 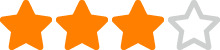	High – Uses an affordance model with a policy to act autonomously based on language instructions	Moderate – Targets specific objects/actions as defined by language input, guided by affordance predictions	Moderate – Generalizes to unseen objects but struggles with complex interactions	High – Decision-making is embedded in the policy, constrained by affordance filtering
Ding et al. (2023) (LLM-GROP) 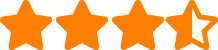	High – Automatically produces task and motion plans for object rearrangement without human oversight	High – Explicitly computes a sequence of symbolic actions to achieve a desired configuration	Moderate – Can generalize to different object arrangements within a symbolic framework	High – Uses hierarchical decision-making with an LLM for planning and a classical planner for execution
Wang et al. (2024b) (LLM^3^) 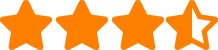	High – Iteratively re-plans based on motion failures without human intervention	High – Never loses sight of the user’s goal; modifies plan to overcome obstacles	Moderate – Handles new object classes via VLM detection, but limited skill set	High – Uses LLM to parse failures and adapts its plan
Stone et al. (2023) (MOO) 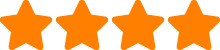	High – Operates autonomously with vision-language assistance for manipulation tasks	High – Identifies target objects based on open-world commands and executes required actions	High – Handles novel objects not seen during training by leveraging broad visual-language knowledge	High – Combines VLM outputs and a learned policy to decide actions in a modular fashion
Lynch et al. (2023) (Interactive Language) 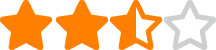	Moderate – Executes segments autonomously but requires continuous human input and confirmation	Moderate – Pursues objectives as defined by the user, though goals may evolve	High – Highly adaptive to human feedback with immediate course corrections	Moderate – Makes decisions iteratively in response to human-provided updates

**TABLE 4:** Agenticness classification for multi-agent and collaborative robotics.

Paper – Overall agenticness	Autonomy	Goal-directed behavior	Adaptability	Decision-making
Mandi et al. (2024) (RoCo) 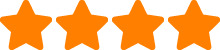	High – Multi-robot system autonomously coordinates tasks via LLM-based dialogue	High – Teams unify around a shared goal	High – Reassigns tasks among robots if one cannot do it, dialogues changes plan dynamically	High – Collaborative discussion” yields joint strategies and path planning in real time
Singh et al. (2024) (MALMM) 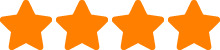	High – Three specialized LLM agents handle planning, coding, supervision autonomously	High – Focus on fulfilling the user’s instructions across multiple sub-steps	High – Adjusts if execution fails by re-planning or re-coding	High – Layered: Planner decides tasks, Coder implements code, Supervisor decides how to fix errors
Wang et al. (2024a) (LaMI) 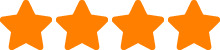	High – Robot autonomously interprets user states and responds	High – Addresses both the functional goal (help user) and social norms	High – Adapts language, gestures, and actions to user’s emotional cues	High – Decides how to respond physically and socially, adjusting communication strategy in real-time
Ahn et al. (2024) (VADER) 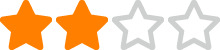	Moderate – Robot proceeds until stuck, then autonomously seeks help from human or other robot	High – Never abandons the mission; actively enlists assistance if blocked	Low – Adapts to unexpected obstacles by weaving in outside help	Moderate – Decides when it cannot solve alone and asks for help, continuing the plan once resolved
Lykov et al. (2023) (LLM-MARS) 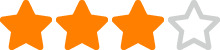	High – Generates behavior trees for multi-robot tasks; no human control	High – Allocates tasks among robots to achieve the operator’s overall objective	Low – Works well within a competition-style environment but domain shift may need re-tuning	High – Plans multi-agent coordination and explains rationale to user
Strobel et al. (2024) (LLM2Swarm) 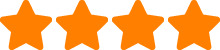	High – Swarm robots can collectively reason or have code synthesized by the LLM	High – They consistently pursue a swarm-level objective, adjusting formation or actions	High – Capable of on-the-fly anomaly handling in the direct integration mode	High – Robots share language-based messages to decide local and global behaviors, showing emergent logic

**TABLE 5:** Agenticness classification for general-purpose multi-task robots.

Paper – Overall agenticness	Autonomy	Goal-directed behavior	Adaptability	Decision-making
Driess et al. (2023) (PALM-E) 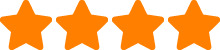	High – Model directly takes sensor input and outputs next actions; no human supervision	High – Conditioned on user goal, it produces step-by-step actions to achieve it	High – Generalizes well to unseen tasks, bridging language and vision	High - Multimodal chain-of-thought lets it integrate scene understanding with goal reasoning
Huang et al. (2022) (Inner Monologue) 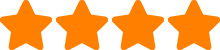	High – Operates autonomously and incorporates feedback to adjust its plan in real time	High – Maintains focus on achieving the final goal by adjusting sub-goals based on outcomes	High – Demonstrates real-time adaptability by revising actions upon detecting failures	High – Uses continuous feedback to re-plan and adjust decisions dynamically
Brohan et al. (2022) (RT-1) 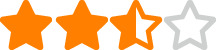	Moderate – Executes tasks autonomously but purely via a supervised policy (no LLM loop)	High – Maps goal descriptions to a series of low-level actions to accomplish tasks	Moderate – Robust on known tasks but limited in adapting beyond extensive training	Moderate – Policy merges vision and language, but decisions are pattern-based, not reasoned in real time
Brohan et al. (2023) (RT-2) 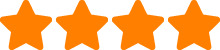	High – Uses a large vision-language model to produce discrete actions in open-ended tasks	High – Consistently focuses on fulfilling user instructions, even if abstract or unseen	High – Adapts to instructions and objects outside its training	High – Embeds decision-making within a deep policy enhanced by fused vision-language features
Reed et al. (2022) (Gato) 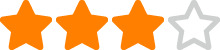	High – Operates autonomously across diverse domains using a single transformer model	Moderate – Pursues task-specific objectives inferred from context, though without explicit planning	High – Adapts to a wide range of tasks via prompt modifications and multi-modal input	Moderate – Uses a learned transformer policy for decision-making without explicit symbolic reasoning
Rana et al. (2023) (SayPlan) 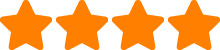	High – LLM handles large-scale environment planning with minimal human monitoring	High – Stays focused on the user’s end goal, divides tasks into multiple room transitions	High – Iteratively re-plans if simulator reports a failure or missing object	High – Scene-graph + LLM synergy yields complex multi-step decisions
Vemprala et al. (2024) (ChatGPT for Robotics) 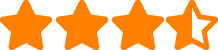	Moderate – ChatGPT can output full plans/code, but guidelines involve human oversight before execution	High – Goal-oriented as it can even ask clarifying questions to refine the goal	High – Adaptable in conversation: if one approach fails, it iteratively re-plans	High – In a tool-using conversation loop, the LLM handles reasoning to plan, correct, and finalize actions
Raptis et al. (2025) (RobotIQ) 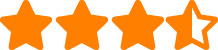	Moderate – Controls tasks autonomously after a single or multiple prompt(s)	High – Focuses on fulfilling user commands from navigation, perception, manipulation	High – Updates or extends plan if environment changes or new instructions arrive	High – Dynamically generates code/ROS calls, deciding the sequence of actions to meet the goal
Wang et al. (2024c) (RONAR) 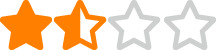	Low – Narrates robot’s actions; does not control them	Moderate – Narration tracks the robot’s progress toward the goal, but does not direct it	Moderate – Narration can adapt to sensor changes; it’s descriptive not transformative	Moderate – Decides how to phrase explanations; does not decide the robot’s physical actions
Ma et al. (2024) (DrEureca) 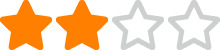	Low – LLM used offline to design rewards and domain randomization, no autonomy at runtime	High – Final RL agent is indeed goal-driven, shaped by the LLM’s reward design	Moderate – Learns across varied sim randomizations, but once learned, no re-adaptation via LLM	Moderate – LLM influences training design decisions; the trained policy does the run-time decision-making

### 3.1 Navigation and robot mobility

Navigation is a fundamental robotic capability–from self-driving cars to home robots–involving understanding high-level goals and translating them into safe paths through complex environments. Traditional navigation requires extensive mapping, path-planning algorithms (e.g., A*, RRT), and often, dense human supervision. LLMs offer a new way to approach navigation by leveraging human-like reasoning about routes, landmarks, and commonsense knowledge of environments. For instance, an LLM can interpret an instruction like “go to the kitchen and then upstairs to the bedroom” and break it down into waypoints or landmark-based steps. Key opportunities related to agentic AI include more natural human-robot communication (using rich language directions) and zero-shot adaptation to complex environments by relying on prior knowledge (e.g., understanding what a “kitchen” typically contains). However, there are notable challenges: i) Spatial reasoning limitations: LLMs lack direct real-world experience, so they may generate paths that are spatially invalid or inefficient. ii) Grounding and perception: The robot must ground the LLM’s high-level plan to its sensors and maps; misalignment can lead to execution failures (e.g., an LLM might “imagine” a straight path that is actually blocked). iii) Real-time adaptation: Dynamic obstacles or changes require reactive adjustments that pure language reasoning might not handle. Recent works address these by combining LLM planning with robotic perception and classical algorithms to ensure physical feasibility. It should be noted that the navigation domain also intersects with other frameworks that use navigation in services like manipulation and others that will be discussed later; here, we focused only on works primarily targeting the navigation problem.

Shah et al. (2023) present LM-Nav, which composes a navigation policy from pre-trained models (a vision-based navigator, CLIP for vision-language grounding, and GPT-3 as the language planner) without any fine-tuning on navigation data. Given a free-form route instruction, the LLM extracts landmarks which are grounded in visual observations by the vision-language model, then a low-level controller navigates to each landmark. Implemented on a real outdoor robot, LM-Nav successfully followed complex multi-turn directions over hundreds of meters, disambiguating landmarks and reaching goals up to 800 m away. The contributions are demonstrating that purely pre-trained models can be assembled for real-world navigation and showing that high-level language understanding can effectively interface with low-level controllers. A key limitation is the reliance on known visual targets (it uses CLIP to match described landmarks) – if the instruction mentions something not visible or known, the system could fail. Nonetheless, LM-Nav pioneered the idea of zero-shot language-conditioned navigation in the real world, validating that an LLM can serve as a route planner when anchored by vision.

Tagliabue et al. (2024) broadens the scope of LLM-driven navigation by emphasizing resilient and adaptive control for Unmanned Aerial Vehicles (UAVs). Unlike standard approaches that focus primarily on route planning or semantic understanding, the authors introduce REAL, an approach for REsilience and Adaptation using LLM to re-tune low-level flight parameters in real time. Specifically, it leverages zero-shot LLM prompting to interpret unexpected failure modes—such as rotor damage or sudden wind gusts—without explicit prior modeling of these anomalies, thereby enabling dynamic mission re-planning. As a result, REAL demonstrates a robust, goal-directed autonomy: the UAV can both maintain its high-level objectives (e.g., surveying a region) and adapt to unforeseen faults by adjusting flight parameters based on LLM recommendations. This higher degree of adaptability stems from bridging commonsense knowledge captured by pre-trained language models with sensor feedback describing flight conditions in textual form. While prompt engineering and validation checks are needed to guard against unsafe suggestions, REAL’s design showcases how LLM-based adaptive control can enhance the resilience of aerial robots toward continuous self-diagnosis and reconfiguration—an essential stepping stone toward agentic, real-world AI deployment in domains like drone navigation.

In navigation, agentic LLMs excel at high-level reasoning and interpreting goals within a rich semantic context as they can infer intent and environmental context in ways classical planners cannot (e.g., recognizing that “find the kitchen” likely means moving through a door and down a hall). The reviewed papers indicate that even without additional training, LLMs carry useful priors for robotics–a form of “embedded common sense” about spatial layouts and routes. Another insight is the necessity of feedback and grounding for true robustness: the most successful implementations use the LLM as part of a feedback loop rather than one-shot output. This is aligned with a broader theme for real-world robotic applications: *LLMs by themselves can plan, but coupling them with real-world feedback vastly improves reliability.*

### 3.2 Manipulation and object interaction

Robotic manipulation–the ability for a robot to pick up, move, and use objects–is central to real-world applications from service robots to industrial automation. It is also a domain where agentic behavior is crucial: a robot must often decide how to grasp an unknown object, sequence sub-tasks (open a jar before pouring, etc.), and handle unexpected outcomes (dropped an item? try again or ask for help). Traditional approaches rely on planning algorithms or learning-based policies trained per task, which struggle with open-world variability. LLMs present an opportunity to drive manipulation with flexible, real-world knowledge planning: they can parse complex instructions, break a goal into steps, and even incorporate commonsense (e.g., knowing you need to hold a cup upright when moving it). This domain has seen a growing interest in using LLMs as high-level planners that interface with low-level motion skills (Cheng et al., 2024).

Despite their strength in reasoning, LLMs lack physical intuition–they do not inherently understand geometry, dynamics, or what sensor inputs mean. This can lead to impractical plans (like pushing an object from the wrong side as a shortcut, which fails in reality (Cheng et al., 2024) or misidentifying objects. Ensuring physical feasibility is a core challenge: many works constrain the LLM’s output by pre-defining low-level skills it can invoke Cheng et al. (2024), Raptis et al. (2025), and Jin et al. (2024). Another challenge is multi-modality: manipulation often involves navigation, vision, touch, etc., so systems must feed these into the LLM or have parallel modules. There are great opportunities, though: LLM-driven manipulation opens the door to generalist robots that can follow human instructions for a variety of tasks (cook a recipe, tidy a room) without task-specific programming. The LLM can also impart “commonsense safeguards” (e.g., do not grip a sharp blade by the edge) if properly encoded, improving safety. In summary, applying LLMs to manipulation is about combining the knowledge and reasoning of language models with the embodiment and experience of robotic controllers. Recent works explore that balance from different angles–planning with code, responding to feedback, learning from demonstrations, etc., - to achieve agentic manipulation.

Ahn et al. (2022) is one of the first to pair an LLM with a robotic affordance model for embodied tasks. The idea is simple yet powerful: use an LLM (Palm 540B) to generate possible next actions from a high-level instruction, but filter those suggestions using a learned value function that predicts which actions are feasible (“affordable”) in the current state. In practice, the robot has a pre-defined set of low-level skills (such as “pick up cup”, “move forward”) and the LLM, given a user request, outputs a sequence of skill suggestions. Each candidate is scored by the affordance model (learned from robot data), and the highest-rated feasible action is executed. SayCan was demonstrated on a mobile manipulator for tasks like “bring me a bottle of water” – the LLM can devise a multi-step plan (go to kitchen, open fridge, grab bottle, etc.), while the affordance filter ensures each step is physically possible (it would not suggest grabbing something that is not there or using a skill out of context). Results indicated significantly higher success rates on long-horizon tasks versus policy baselines, and the system could handle over 100 instructions in a household setting. A limitation is dependency on the set of pre-implemented skills–SayCan cannot invent truly novel actions, it can only compose the provided ones. Also, the affordance model must be trained for each new skill or environment. Nonetheless, SayCan established a template for embedding agency in robots by having the LLM reason over actions and the robot veto or execute, ensuring safety and feasibility.

On the other hand, Singh et al. (2023) proposes ProgPrompt, a structured prompting method to improve LLM planning for embodied agents. Instead of giving the LLM a raw text instruction and letting it free-form, they prompt it with a program-like specification of the environment and actions. For instance, they describe the available objects and actions in a pseudo-code format and provide a few examples of plans as small programs” (like a sequence of function calls or steps). The LLM then generates a plan in that programmatic format, which can be directly executed by the robot’s controller or easily parsed. The key insight is that by shaping the prompt as a programming problem (with structure and examples), the LLM’s output becomes more reliable and unambiguous for situated tasks. They tested it in household tasks (in simulation) where the robot had to move objects around a kitchen based on high-level directions. ProgPrompt’s LLM (GPT-3) produced correct and efficiently structured plans more often than a baseline prompting method, especially as tasks grew more complex. The plans also generalized across changes in the environment (like if an object was not where expected, the LLM could include a search step). This work contributes a methodology to reduce the hallucinations” or omissions in LLM-generated plans by giving the LLM a sort of formal language to work in. It echoes the theme of constraining the LLM with structure to get safer, more predictable behavior. Limitations include the need to manually define the domain specification (you must list all possible actions/objects in the prompt, which might be hard in an open world), and scaling to very large environments could make prompts unwieldy. However, it provides a bridge between classical planning languages and LLM flexibility.

Jin et al. (2024) propose RobotGPT, a framework that uses ChatGPT as a “teacher” to train a more stable robotic policy, addressing the unpredictability of directly deploying an LLM for manipulation. The authors observed that code generated by ChatGPT for robot tasks can be inconsistent (different outputs for the same prompt) and sometimes unsafe or unstable. To leverage ChatGPT’s problem-solving strengths without these downsides, RobotGPT generate diverse solution codes for manipulation tasks (e.g., pick-and-place scripts), and then uses those as synthetic demonstrations to train a student model. Essentially, ChatGPT’s knowledge is distilled into a policy network that is less prone to randomness and can be executed reliably on the robot. In both simulation and real-world evaluations, RobotGPT achieved a dramatic improvement in task success rates–e.g., averaging 91.5% success versus 38.5% when using ChatGPT-generated code directly, highlighting the key finding that a trained policy (even one trained on LLM-produced “advice”) is far more repeatable and safer than calling the LLM at runtime. Limitations of the approach include the need for a sufficiently rich set of LLM-generated solutions. Additionally, the student policy’s performance is bounded by the quality of the LLM “expert”; if the LLM’s outputs are suboptimal, the policy will reflect that (though training can average out some errors).

Duan et al. (2024) introduce a system for automating general robotic manipulation by leveraging vision-language models (VLMs) to generate action trajectories without hand-crafted skills or privileged simulation states. Manipulate-Anything uses a multi-phase pipeline: first, given a scene (via multi-view images) and a natural language task, a VLM identifies the relevant objects and suggests sub-tasks needed. For each sub-task, an action generation module computes a concrete action execution–such as a 6-DoF end-effector pose or a parameterized motion code–guided by the VLM’s understanding of affordances and goals. A verification module then checks via vision whether the sub-task was successful; if not, the system can recover by reattempting or adjusting the action.

Liang et al. (2023) takes a different approach to LLM-driven manipulation: instead of outputting plain-language plans, the LLM generates executable code that serves as the robot’s policy. Here, the authors prompt the LLM with a few examples of high-level instructions paired with Python code that calls primitive robot APIs (functions for moving arms, gripping, etc.). Given a new instruction, the LLM writes new code by composing those API calls (and even using libraries for calculations). The result is essentially a programmatic plan: e.g., for “push the red block to the green area,” the LLM might generate a code sequence that queries an object detector for “red block,” computes a path to the green area, then calls a motion primitive to push in small increments. A major benefit of this approach is transparency and verifiability: the output is code that a human can inspect or simulate before executing on the robot, adding a safety layer. The experiments on real robots (manipulating blocks, etc.) demonstrated that many tasks could be achieved without additional training, just via few-shot prompting of the LLM. Challenges include ensuring the generated code is safe (the LLM might still produce code that causes erratic movements if not constrained) and dealing with execution errors–if the LLM writes a bug or the robot deviates, there must be a mechanism to recover. Overall, Code-as-Policies is a compelling demonstration of an LLM acting as a high-level policy programmer, merging symbolic AI (classical programming) with data-driven language understanding.

Bousmalis et al. (2023) propose RoboCat, another multi-task, multi-embodiment agent, but with a focus on continual learning. It starts by training a vision-based decision transformer on data from a few types of robotic arms doing many tasks. Then, crucially, it demonstrates the ability to adapt to a new robotic arm and new tasks by fine-tuning on a new setup. After fine-tuning, RoboCat uses that agent to generate more data on the new tasks through self-play, adds that to its corpus, and retrains the foundation model (hence “self-improving”). Over iterations, RoboCat gets better and can handle an expanded set of tasks and new arm morphologies with minimal human data each time. Authors showed it could learn to control a new robot arm with different gripper in a new task with very little new data, and each retraining phase increased its overall skill set without forgetting old ones. This is significant as one vision for a generalist agent is the one that grows and learns over its lifetime, not just a static trained model. Limitations include large compute for retraining each time and the domain still being manipulation-centric (it does not do language or navigation, etc.). But it is a template for how an agentic robot might learn like an animal–gradually increasing its repertoire by interacting with the world and consolidating that experience.

Huang et al. (2023) by introducing VoxPoser, addresses the challenge of grounding LLM plans in continuous 3D space, aiming to remove the need for predefined motion primitives. In simpler terms, given an instruction like “open the top drawer,” the system uses a pre-trained VLM to parse the instruction and scene image to identify where the action should apply (e.g., the handle of the top drawer). It then constructs a 3D voxel map (in the robot’s coordinate frame) with values indicating the desirability of moving the end-effector to each location–a sort of goal heatmap. A motion planner uses this “value map” to generate a trajectory to the high-value region and execute the action. By composing multiple such value maps in sequence, VoxPoser can perform multi-step tasks. Authors demonstrate that an LLM+VLM can effectively output continuous action targets (not just discrete steps) in a zero-shot way, enabling the robot to do things it was never explicitly trained to do by following language-informed hints. For example, if told “I am left-handed” during a table-setting task, the system can adjust the value maps to place utensils on the left side of plates (the LLM/VLM interprets this preference and alters the target positions). This showcases a high degree of adaptability and semantic understanding in manipulation. This approach improved success on tasks like pushing and object reorientation as it allowed flexible motion generation rather than relying solely on fixed primitives. However, VoxPoser requires good calibration between vision and robot coordinates, and errors in the value map could cause poor trajectories. In summary, VoxPoser pushes agentic manipulation further by letting the LLM effectively paint a target for the robot in 3D space, blending symbolic language goals with continuous control.

An alternative approach to bridging language and action is to leverage visual affordances to constrain decision-making. In this direction, Mees et al. (2023) tackles the problem of connecting language instructions to actionable perceptions in an unstructured environment. The authors propose HULC++, using a visual affordance model (trained on “play” data) to suggest what interactions are possible with objects in the scene and then using those as conditioning for an instruction-following policy. For example, if the command is pick up the toy on the couch,” the affordance model (which has learned from unlabeled play data how objects can be grasped or moved) highlights the toy as graspable and perhaps where to grasp it. The result is improved sample efficiency as the affordances restrict the action search space to likely successful ones. HULC++ is showing that unstructured play data (random explorations by the robot) can be leveraged to create a grounding mechanism for language–a practical way to handle novel objects. In their experiments, the combination of language + affordance outperformed policies that relied on language or vision standalone tasks. One limitation is that the affordance model might not cover very complex interactions (like using a specific tool) unless such data was in the trained dataset. Nonetheless, this method points toward self-supervised grounding–robots learning from their own experience how to interpret language in terms of what can be done in the real-world.

Ding et al. (2023) introduces LLM-GROP, which integrates LLMs into classic Task and Motion Planning (TAMP) for object rearrangement tasks. The LLM is used to generate a high-level symbolic plan (sequence of discrete actions like “pick A, place on B, then grasp C”) from a language instruction, and then a motion planner computes the continuous trajectories for each action. Essentially, it replaces the task planner with an LLM that can parse general instructions and output actions in Planning Domain Definition Language (PDDL)-like form (Haslum et al., 2019). The novelty is that the LLM can incorporate commonsense constraints or preferences directly from the instruction Results on object rearrangement tasks showed that the LLM-planned sequences were valid and often more efficient than baseline TAMP planners. A challenge was ensuring the LLM’s output was parseable and correct for the motion planner–they had to do prompt engineering and post-checks to avoid nonsensical actions. LLM-GROP’s contribution lies in marrying LLMs with TAMP: leveraging the LLM’s flexibility to generate plans for open-world goals while still using proven motion planning for execution.

Similar to LLM-GROP, Wang S. et al. (2024) proposes LLM^3^ which also integrates an LLM into classical TAMP. Here, the pre-trained LLM serves as a universal task planner that suggests symbolic actions and even continuous parameters for a motion planner, i.e., grasp positions, Crucially, LLM^3^ runs in an iterative loop: if the motion planner fails (e.g., a path is obstructed or a grasp is invalid), it feeds that feedback to the LLM via prompting, allowing the LLM to reason about the failure and adjust the plan. This closed-loop reasoning markedly improved success rates in simulated box-packing tasks, and the authors demonstrated the approach on a real manipulator arm, confirming its practicality in physical settings.

Stone et al. (2023) is enabling manipulation of objects beyond the robot’s training distribution by using pre-trained vision-language models (VLMs). The approach, called MOO (Manipulation of Open-World Objects), uses a VLM (trained on internet-scale image-text data) to extract object identity and relevant features from a camera image and a language command. For example, if asked “pick up the spork,” the VLM can identify which object in the scene is a spork (even if the robot has never seen one in training) and provide an embedding for “spork”. This information is then used to condition a learned manipulation policy that was trained on broad data but with generic object representations. The main contribution is a system that interfaces learned manipulation policies with a frozen VLM to achieve open-vocabulary object manipulation. In trials, the robot could execute commands involving novel object categories by relying on the VLM to point it to the right object and sometimes even suggest grasp points via visual cues. This extends the robot’s capabilities without additional robot training on those objects–effectively *transferring knowledge* from internet data to robot actions. The outcome is moving toward generalist manipulation where the bottleneck of limited object categories is removed. Limitations include dependence on the VLM’s accuracy–if the VLM fails to recognize the object or confuses it, the policy is conditioned on wrong info. This work aligns with the general trend of using foundation models to expand robotic perception and reasoning, here applying it to achieve a greater breadth of object understanding in manipulation tasks.

Beyond leveraging vision-language models for object recognition and manipulation, another challenge in robotic task execution is ensuring that LLM-generated plans are structured and interpretable. Singh et al. (2023) in ProgPrompt proposes a structured prompting method to improve LLM planning for embodied agents. Instead of giving the LLM a raw text instruction and letting it free-form, they prompt it with a program-like specification of the environment and actions. For instance, they describe the available objects and actions in a pseudo-code format and provide a few examples of plans as small “programs” (like a sequence of function calls or steps). The LLM then generates a plan in that programmatic format, which can be directly executed by the robot’s controller or easily parsed. The key insight is that by shaping the prompt as a programming problem (with structure and examples), the LLM’s output becomes more reliable and unambiguous for situated tasks. They tested it in household tasks (in simulation) where the robot had to move objects around a kitchen based on high-level directions. ProgPrompt produced correct and efficiently structured plans more often than a baseline prompting method, especially as tasks grew more complex. Singh et al. (2023) proposed a methodology to reduce the “hallucinations” or omissions in LLM-generated plans by giving the LLM a sort of formal language to work in. Limitations include the need to manually define the domain specification (you must list all possible actions/objects in the prompt, which might be hard in an open world), and scaling to very large environments could make prompts unwieldy. However, it provides a bridge between classical planning languages and LLM flexibility.

Another key challenge is to enable robots to adapt their actions dynamically through human interaction. Interactive Language (Lynch et al., 2023), allows humans to give incremental instructions and corrections via dialogue to an LLM-controlled robot. For example, as a robot arm is stacking blocks, the user might say, “actually, put the blue block on the red one instead” – the LLM parses this mid-course correction and alters the plan on the fly. The contribution of this paper is mainly in demonstrating the feasibility of fluent back-and-forth communication with a robotic manipulator. In their experiments, users were able to iteratively guide the robot through complex arrangements by conversation, achieving goals that would be hard to specify upfront. This approach leans heavily into the agentic property of adaptability–the robot is not just executing a fixed plan; it is reacting to human inputs continuously, effectively sharing control. The challenges include maintaining coherence in the dialogue (the LLM must remember prior instructions and the current context) and timing (ensuring the physical robot’s actions stay synchronized with the dialogue–not executing outdated commands). While not introducing new planning algorithms, this work underscores an important aspect of agentic AI in robotics: interactive guidance and collaboration, where the “agent” is not an isolated decision-maker but part of a team with a human. Last but not least, it also raises interesting implications for safety–a human can step in and correct a mistake verbally, potentially avoiding failures. In summary, Interactive Language shows that an LLM-enabled robot can be treated almost like a human assistant that you can talk to and supervise in real time, marking a move toward natural human-robot collaboration.

Across manipulation works, we see a spectrum from high-level planning to low-level control, and different ways of injecting agentic behavior. The fusion of LLMs with robot manipulation has revealed that language-based reasoning can dramatically improve multi-step task performance in unstructured settings. One insight is that LLMs provide a form of “transfer learning” – they bring a wealth of commonsense (e.g., knowing tools, typical order of actions) which allows robots to perform tasks with minimal or no task-specific training. This is evidenced by successes like assembling simple structures by following written instructions or handling objects never seen before. Another insight is the importance of grounding and feedback for true agentic behavior. Systems that treat the LLM as a continuously observing and updating agent (rather than a one-shot planner) achieve far greater robustness. This mirrors human problem-solving: we do not just execute a plan blindly; we check and adjust. By giving LLMs a chance to do the same, robots can behave more “agentically” and can cope with surprises and partial information in real time.

### 3.3 Multi-agent and collaborative robotics

Many real-world scenarios include multiple robots working in coordination. In such settings, communication, coordination, and division of tasks become as important as individual task execution. Agentic AI in a multi-agent context means each AI entity not only plans for itself but also interprets others’ actions, communicates intentions, and possibly negotiates or assists. LLMs are a natural fit for the “communication” aspect: they understand and generate language, which can serve as the medium of coordination. We are seeing LLMs used as controllers or mediators in multi-agent systems, effectively bringing the power of natural dialogue and reasoning into group settings.

However, multi-agent settings compound the usual difficulties. Communication can be a double-edged sword: misunderstandings between agents (even AI ones) can lead to failures. Ensuring a shared mental model (common knowledge) is tricky. From a safety perspective, coordinating multiple effectors can be dangerous if not done carefully (e.g., two robotic arms moving in the same space). There is also the question of scalability–an LLM orchestrator might handle a few agents, but does it scale to swarms of 100 robots? Another challenge is real-time performance: multi-agent interactions often need timely responses (a delay in communication can ruin coordination), and LLMs, especially large ones, can be slow. Additionally, in human-robot collaboration, understanding human intent (possibly from ambiguous dialogue or partial instructions) and maintaining trust are important–the AI must know when to yield control or how to explain its suggestions. Despite these, opportunities abound: multi-agent LLM systems can bring flexibility in how we deploy robot teams. They also allow mixing different types of agents (a vision system, a robot arm, a drone, a database) because language -as we already know as humans- is a universal interface.

One approach is using a centralized LLM agent that plans for all robots. RoCo (Mandi et al., 2024) is an example of a dialectic multi-robot collaboration framework: a single large language model is prompted with the goals and observations of two or more robots and is tasked with outputting coordinated instructions for each robot. For instance, if two robots must clean a house together, the prompt to the LLM may describe Robot A’s view (e.g., it sees a dirty kitchen) and Robot B’s view (a messy living room) and ask for a plan. RoCo’s LLM might respond with a detailed strategy like: Robot A: start cleaning the kitchen counters; Robot B: vacuum the living room; once done, both meet to take out trash.” The contribution of this approach is showcasing that a single LLM can implicitly perform task allocation and scheduling, leveraging its knowledge to balance workloads and sequence tasks logically. Experiments in both simulation and real-world, demonstrated successful coordination without explicit symbolic planning–the language model essentially writes the playbook for the team. However, a limitation is scalability: as the number of agents or the scenario complexity grows, a single LLM context may become too large (or the prompt too complicated), leading to degraded performance or hitting token length limits.

Shifting toward purely robotic teams, MALMM (Singh et al., 2024) introduces a framework where multiple LLMs, each with a specialized domain (e.g., path planning or grasp selection), communicate to solve zero-shot tasks such as joint assembly. By distributing responsibilities, MALMM avoids overloading a single model, and inter-LLM negotiations can produce more refined solutions. However, the overhead of coordinating multiple language models can become significant, and misalignments in knowledge among them may produce contradictory subgoals. A broader multi-modal angle emerges in Wang C. et al. (2024), which tackles human-robot interaction involving speech, gestures, and visual cues. In LaMI, the LLM acts as the central orchestrator, fusing these diverse inputs into a shared textual representation. This design enables robots in group assembly tasks to interpret partial verbal commands plus a pointing gesture or a head nod and respond accordingly, illustrating the integrative power of language-based frameworks. However, real-time multi-user settings pose scaling issues, as more complex dialogues require advanced conversation management.

Introducing error recovery via visual signals, VADER (Ahn et al., 2024) is a framework enabling robots to proactively seek help from humans or other robots when facing failures in long-horizon tasks. The system works in a plan–execute–detect loop: it first uses an LLM to generate a task plan from the instruction. As it executes each step, visual question answering (VQA) modules continuously monitor for anomalies or affordance issues–for example, checking if the action had the intended effect or if an object needed for the next step is missing. If a robot fails to complete a task, the system checks whether another robot or a human can execute that step, swiftly reallocating the task. VADER’s major contribution is formalizing “seeking assistance” as part of the plan output by an LLM, rather than as an *ad hoc* external intervention. This dynamic fallback mechanism underscores the advantage of an LLM that can interpret real-time sensor data and reason about which agent is best suited for the subtask. However, like many collaborative frameworks, consistent multi-view perception and accurate affordance detection remain potential bottlenecks.

An alternative approach is introduced by Lykov et al. (2023). They propose LLM-MARS, a system that integrates an LLM into a multi-robot team to handle both strategic planning (via behavior tree generation) and interactive dialogue-based supervision When the operator gives a command to a team of robots, the LLM-MARS will output a behavior tree that allocates tasks among the robots and sequences their actions logically to achieve the goal. For instance, if a robot is blocked, a quick textual request triggers the LLM to alter relevant behavior tree nodes. This fosters flexible adaptation and transparency into a multi-robot system: the human can issue complex commands in NLP and get both effective task completion and clear verbal justifications; however, it underscores the importance of verifying that automatically generated tree modifications remain consistent and avoid deadlocks.

Last but not least, Strobel et al. (2024) introduce LLM2Swarm which examines larger-scale swarms, wherein an LLM suggests global swarm strategies and each agent partially consults that blueprint for local decisions. The authors outline two integration approaches: (1) Indirect, where an LLM is used off-line to write or verify swarm controller code and (2) Direct, where each swarm robot runs a local instance of an LLM in real-time, enabling the robots to communicate in natural language and reason on the fly during missions. In both modes, the swarm can collaboratively adjust formation or plan using language as an intermediate representation. Proof-of-concept showcases demonstrated robots detecting anomalies that were not pre-specified (like an unexpected object on the field) and coordinating a response in a human-like manner, all without explicit anomaly-handling code. A key limitation in such systems, is scaling to hundreds or thousands of agents may create severe communication overhead, pointing to a need for hierarchical communication to keep LLM prompts manageable in real-time applications.

Across these works, the collaborative use of LLMs reveals that language is a powerful tool for coordination. An important insight is that many AI and robotic components can be connected with near-zero integration effort by using language as the intermediary. Another insight is the concept of dynamic autonomy: agentic systems do not have to be all-or-nothing autonomous; the best outcomes often involve an agent reasoning about when to take action independently and when to consult the others. Ren et al. (2023) formalizes that intuition by giving statistical guarantees, essentially teaching the AI that sometimes asking for help is the smartest thing you can do. This is a profound shift from earlier AI, which often either operated autonomously or relied on scripted human intervention–*now the AI itself decides when and how to include humans*.

### 3.4 General-purpose multi-task robots

General-purpose multi-task robots represent the “holy grail” of robotics and AI–agents that can perform a wide variety of tasks across different domains (locomotion, manipulation, perception, language) without being re-designed for each new job. Historically, robots and AI systems have been narrow: excellent at one task, clueless outside that niche. The recent rise of foundation models (huge models trained on broad data) suggests a path to generalist robots. However, to truly achieve general-purpose agency, several critical challenges must be addressed. First, data collection and training costs remain a major bottleneck—training such generalists requires vast and diverse datasets, which are often prohibitively expensive to acquire. Second, there is the issue of embodiment mismatch: while LLMs possess vast world knowledge, they often lack understanding of the specific dynamics of physical robots. Third, evaluation remains an open problem—these systems may not outperform specialized models on single benchmarks, yet they demonstrate impressive versatility across domains, calling for more holistic metrics of success. Fourth, concerns around safety, forgetting, and robustness persist; fine-tuning for new tasks risks erasing prior capabilities, and ensuring safe behavior in unfamiliar contexts is an ongoing challenge.

PaLM-E (Driess et al., 2023) opens this category by combining vision, language, and action in one model to allow richer understanding of tasks. PaLM-E is essentially Google’s large language model PaLM (which has 540B parameters) extended with embodied sensor inputs–specifically, images and continuous states are encoded and fed into the model alongside text. The result is a colossal 562B parameter model that can take in an observation (like an image from a robot’s camera) and a command, and output a plan or action commands in text form. Trained on a combination of internet-scale language data and robot-specific multimodal data (from multiple embodiments, e.g., mobile robots and manipulators), PALM-E can perform a variety of embodied reasoning tasks–sequential planning for manipulation, visual question answering about the scene, and even fuse the two. Notably, it shows positive transfer: training on multi-task multimodal data improved performance on each individual task compared to training separate models, indicating synergy between skills. Limitations are its size (currently impractical for real-time on-robot use) and the fact it was only tested in relatively structured environments and simulation for robotics tasks. However, PaLM-E marks an important step: an existence proof that a beefy language model can digest real-world inputs and act in a grounded way across tasks.

Another outstanding approach was introduced by Huang et al. (2022). Inner Monologue extends the LLM-as-planner concept by incorporating explicit language feedback loops during execution. In this framework, the LLM does not just output a static plan; it continually updates its plan (its “inner thoughts”) based on feedback such as success/failure signals, visual observations, or even Q&A with a human. The authors introduce various feedback types: passive scene descriptions (automatic observations like “object X moved”), active queries (the LLM can ask a vision model or human a question if uncertain), and success detection (binary signals of whether the last action achieved its intended effect). Experiments on a real robot in a kitchen space showed that this closed-loop approach significantly improves success rates over open-loop LLM plans. For example, if the instruction is to set a table, the LLM might plan to place a plate and then a cup. If the cup placement fails (detected by vision), the LLM’s inner monologue incorporates “the cup fell, try a different grasp” and adjusts the plan. This ability to react dynamically to intermediate real-world feedback makes Inner Monologue a compelling demonstration of how LLMs can function as self-reflective agents that behave more autonomously and safely, leading to more robust and adaptable robotic performance. However, one challenge is the complexity of prompt engineering–the LLM needs a structured prompt that includes its action history and new observations each cycle. Poorly designed prompts risk the LLM misinterpreting or overlooking crucial feedback. Another limitation is reliance on the quality of external feedback (if vision mis-describes a scene, the LLM might be led astray). Yet, Inner Monologue is a key step toward interactive, situationally aware robot planners using LLMs, effectively imbuing the robot with a form of introspection and adaptability mid-task.

Another avenue for achieving versatile robotic behavior is training policies on extensive real-world demonstrations. Brohan et al. (2022) propose RT-1, an example of training a general policy on a large-scale robot dataset. The authors collected 130k+ demonstrations (over 700 tasks) with a fleet of robots–mostly household and manipulation tasks. Thanks to the massive and varied dataset, RT-1 achieved robust performance on tasks like picking, placing, opening drawers, etc., and even showed zero-shot generalization to some new instructions which they reported 97% success on seen tasks and 76% on unseen tasks. However, RT-1 lacks explicit reasoning or modularity–it is like a large implicit library of behaviors. Despite the large training set, RT-1 is limited to the distribution of its data. It may fail with completely novel objects or instructions containing words it has not seen.

On the other hand, RT-2 (Brohan et al., 2023) builds on RT-1 but pushes generalization further by incorporating web-scale vision-language data into the training. Instead of training from scratch on robot data, the authors co-fine-tune a large model on two types of data simultaneously: (a) the RT-1 robot demonstration dataset (actions labeled with instructions and images), and (b) internet-scale vision-language datasets. Crucially, the authors unify the output format by treating robot actions as another “language”. They map each discrete action token (from RT-1’s vocabulary) to a text string, allowing actions to be appended to the same training corpus as language sentences. For example, an image with caption “A person holding a bowl” might be in the training mix, and an image with an instruction “pick up the bowl” would be paired with output tokens representing the pick action. The model (a Vision-Language-Action transformer) learns to produce either natural language or action sequences as appropriate. Results showcased that RT-2 is one of the first works to show that we can transfer internet knowledge into direct robotic actions effectively. A challenge with RT-2 is complexity: these vision-language models are huge (though smaller than PaLM-E’s full LLM), and integrating them requires careful fine-tuning so as not to ruin the pre-trained features. It also still relies on the quality of both web data (which might have biases) and robot data. However, RT-2 moves closer to the dream of a robot that “knows what the internet knows” and can act on it.

Talking about general-purpose multi-tasking, Reed et al. (2022) introduce Gato: “A Generalist Agent”. Gato from DeepMind is a landmark model showing a single transformer can handle totally different domains. The idea is to use one model (with one set of weights) to control an agent in text-based dialogs, vision-based games, and robotic control alike. Thus, the authors trained Gato on a mix of data: images and actions from Atari games, text dialogues, robot arm trajectories, etc. The impressive result was that with one set of weights, Gato showed competence ( > 50% expert score) on 450+ tasks out of 604 after training. These included controlling a real Fetch robot arm to stack blocks, playing Atari games at a decent level, captioning images, chatting about general topics, and more. Gato is a proof-of-concept that *unification is possible*–that the diversity of sensorimotor and language tasks can be addressed by one architecture.

Another key dimension is the extent of environmental knowledge. Rana et al. (2023) introduce SayPlan which fuses an LLM with a 3D Scene Graph (3DSG) of the environment as an intermediary world model for the LLM. Specifically, the scene graph is a structured representation of rooms, objects, and their connectivity (e.g., a graph node for each room and object). The LLM queries the 3DSG for spatial relationships (e.g., “Is there a doorway between the living room and the kitchen?”) to produce elaborate multi-step plans for large-scale tasks. Authors demonstrate that hooking an LLM to a structured world model extends planning beyond single-scene or single-step manipulations. Although it is capable of discovering efficient routes and object-handling sequences, reliance on a pre-built or updated scene graph remains a central bottleneck in truly dynamic settings.

Vemprala et al. (2024) proposes ChatGPT for robotics. This Microsoft report applies ChatGPT to control robots through dialogue and reasoning, focusing on design principles and model abilities. One key aspect is iterative prompting and verification: the approach involves having the LLM first produce a high-level plan, followed by either refinement through additional prompts or human verification before proceeding. Afterward, the LLM can generate specific robot code if required, continuing the process as needed. This effectively creates a multi-agent loop between the human, the LLM, and the robot where the LLM sometimes takes the role of an advisor and sometimes as an executor. Their contributions lie on: (1) Highlighting the importance of model self-awareness of uncertainty–ChatGPT can be prompted to express uncertainty and suggest asking a human (aligning with “robots that ask for help”), (2) Demonstrating a variety of robot control examples (drones, arms, home robots) all through one interface: ChatGPT. While ChatGPT for robotics is not a single unified system, this work shows that with the right prompts and safety checks, ChatGPT can successfully handle multi-step instructions for robotics and interact with a user to clarify goals. It essentially treats the human and ChatGPT as a collaborative pair jointly operating the robot where the human provides high-level intent and oversight and ChatGPT provides detailed reasoning and translation to code or robot API. A challenge is ensuring ChatGPT does not produce unsafe instructions; they recommend heuristic filters and user confirmation for critical actions. The general message is that conversational LLMs are promising agents” to mediate between user and robot, plan tasks, and even coordinate multiple actions. It encourages a paradigm where you can chat with your robot’s AI to get things done, analogous to how you would coordinate with a human co-worker.

Similar to the previous, Raptis et al. (2025) introduce RobotIQ, which proposes a full-stack system where a robot can leverage any LLM (GPT-4, etc.) to interpret user commands and generate a task plan, which is then executed via a ROS (Robot Operating System) pipeline. The architecture has modular components: (a) Natural Language Interface: The user can give instructions by text or voice. The LLM receives this instruction along with context (robot’s capabilities, current world state) in a prompt. (b) Robotic API Library: Similar to ChatGPT-for-Robotics, they define a library of ROS actions and services (like “NavigateTo(location)”, “FindObject(name)”, “PickUp(object)”) that the LLM can call. (c) Execution and Knowledge Transfer: RobotIQ emphasizes transferring knowledge from simulation to reality acting as a bridge between high-level reasoning and low-level ROS controls, all mediated by an LLM “brain”. The author’s contributions lie on: (1) Integrated System in ROS: It is one of the first frameworks to show end-to-end integration of an LLM with ROS, making it practical for ROS developers to add language intelligence. They release an open-source RobotIQ library with ready-to-use ROS modules and prompts, so one can plug in an LLM and get a functional system. (3) Human-level planning with common-sense: The use of a powerful LLM endowed the robot with some common-sense reasoning. Evaluations in simulation and real-world settings show that RobotIQ can efficiently handle multi-step tasks, underscoring the potential of the Robot-as-a-Service (RaaS) model while incorporating human feedback and domain-specific knowledge into its prompt-engineering processes.

Wang Z. et al. (2024) propose RONAR, an LLM-powered system designed to generate natural language narratives based on a robot’s sensory and internal data for transparency and facilitating failure recovery. The system processes multimodal inputs—such as joint motions, camera images, force sensor readings, and discrete events—by first converting them into high-level contextual summaries using heuristics or lightweight models (e.g., anomaly detection or keyframe extraction). These summaries are then fed into an LLM, which produces concise descriptions of the robot’s experiences, such as “I tried to pick up the block, but I dropped it”. Its contributions include grounding complex sensor data into human-understandable language, improving failure detection and response time (users intervened 1.5x faster when narratives were present), and providing a real-world dataset of annotated robot episodes. While the LLM does not control the robot’s physical actions, it exhibits a form of metacognitive agency by autonomously generating observations and suggestions, effectively acting as the robot’s “inner voice”. RONAR’s domain-agnostic design was demonstrated across scenarios including long-horizon mobile manipulation and tabletop tasks, covering both nominal operations and failures. RONAR opens up opportunities for tighter integration with instruction-following systems, customized narrations based on user expertise, and the use of self-narration as a supervisory signal for training future policies—pushing forward the role of agentic AI in human-robot collaboration.

Ma et al. (2024) introduces an LLM-guided framework that simplifies sim-to-real reinforcement learning by automating two traditionally manual and expertise-driven processes: reward function design and domain randomization. By prompting a large language model (GPT-3) with natural language descriptions of task goals, DrEureca generates reward function code or pseudocode and suggests relevant simulation parameters to randomize (e.g., friction, motor torque, sensor noise). These LLM-derived configurations are used to train policies in simulation, which are then deployed to real robots. The framework reduces the need for expert intervention and achieves performance comparable to hand-crafted setups, even enabling success in previously unsolved tasks, purely from LLM-suggested rewards. While the LLM does not control robot actions directly, it plays an agentic role during the training design phase, acting as an AI assistant to human developers. Validated across quadruped locomotion and dexterous hand manipulation tasks, DrEureca demonstrated sim-to-real transfer on physical robots. Limitations include sensitivity to prompt quality, potential for reward hacking or omission of safety constraints, and a reliance on human input for iteration if trained policies underperform. Overall, DrEureca points to a future where specifying robot learning tasks in natural language becomes a practical and powerful alternative to manual engineering.

In essence, the progress in this domain suggests that robotics might follow a trajectory similar to NLP: moving from task-specific models to a few large general models that can be adapted to myriad tasks. Agentic AI behavior here is about being able to set and pursue goals in virtually any context, given the right prompting or minimal experience–a hallmark of higher-level autonomy. We are not there yet, but these works provide a roadmap. They indicate a future where robots have a kind of general intelligence substrate (an LLM or transformer-based policy) that can be directed to tasks via goals or instructions, much like we humans apply our general intelligence to whatever task is at hand.

Overall, examining patterns by task domain, we find that different applications emphasize different facets of agenticness. In Navigation and Mobility tasks, LLM-equipped robots tend to excel in Autonomy: once given a destination or instruction, navigation robots can independently chart routes and traverse their environments without step-by-step human guidance. Their Decision-Making requirements, however, are often more constrained–navigating largely involves spatial decisions (choosing paths, avoiding obstacles) which, in many implementations, are assisted by map-based planners or reactive policies. In Manipulation and Object Interaction, systems typically demonstrate very strong Goal-Directed Behavior and notable Adaptability. Each manipulation task has a clear goal (like assembling a part or retrieving a specific item), and the robot must often handle variability in objects and environment. Consequently, these works highlight the LLM’s ability to adapt plans on the fly–for example, a robot arm might figure out how to open an unfamiliar drawer mechanism to find a target object, or adjust its grasp if an object slips, all in service of the specified goal. In Multi-Agent and Collaborative Robotics, the dominant theme is sophisticated Decision-Making through communication and dynamic role allocation. Here, an LLM or a network of LLMs facilitates coordination among multiple robots, often by having agents communicate in natural language to share information and divide responsibilities. This leads to very agentic group behavior: the robots collectively decide who will do which sub-task and when to assist each other, guided by the LLM’s high-level reasoning about the team’s objectives. Finally, the Generalist Multi-Task Robots exhibit the broadest agenticness across all dimensions. These systems, built on powerful foundation models, integrate multiple modalities (vision, language, action) and can perform a wide range of tasks, requiring high Autonomy, goal-driven flexibility, and context-aware decision-making all at once. In the surveyed papers, such generalist robots leverage LLMs to plan and execute across navigation, manipulation, and more within a single unified framework. As a result, they must achieve a balance of skills: strong autonomy to handle open-ended missions, adaptive reasoning to generalize across diverse scenarios, and careful decision-making to select the right tools or actions for each robotic task.

## 4 Ethics, safety, and transparency

LLM-driven robotic systems must not only perform tasks but do so in a manner aligned with ethical norms, safety requirements, and principles of transparency. This section examines how recent systems incorporate (or overlook) these considerations. We first define key Ethical, Safety, and Transparency Metrics (Section 4.1). We then group the surveyed systems into clusters based on how strongly they emphasize these metrics (Section 4.2) and finally analyze trends in different task domains (navigation, manipulation, multi-robot collaboration, and general-purpose robotics) using a scoring framework (Section 4.3).

### 4.1 Ethical, safety, and transparency metrics

To evaluate LLM-based robotic systems on ethics, safety, and transparency, we consider several metrics that emerge from the literature and by taking into consideration EU AI Act (Regulation (EU) 2024/1689)^1^. Below we define each metric and provide examples from representative reviewed items.

• Fairness and Bias: Assessing whether an LLM-driven robot’s decisions or outputs exhibit unfair prejudice or stereotypes towards certain groups is crucial. Bias detection typically involves analyzing model behavior across demographic or contextual variations to uncover systematic favoritism or discrimination (Azeem et al., 2024). For example, studies have found vision-language models in robots less frequently recognize certain races or genders in specific roles (Hundt et al., 2022). Detecting such biases is crucial to ensure robotic assistants treat all users and scenarios equitably and do not perpetuate harmful stereotypes. The EU AI Act mandates that high-risk AI systems, such as those used in employment or education, ensure the quality of datasets, including their relevance, representativeness, and absence of bias, to prevent discrimination (Article 10).

• Safety Guardrails: Technical and procedural measures that prevent robots from causing physical or psychological harm. These guardrails constrain the LLM’s behavior to a safe operational envelope, for instance by filtering or modifying potentially dangerous commands and actions (Dong et al., 2024). Intuitively, guardrails act like a safety net–monitoring the robot’s inputs and outputs and intervening if an instruction could lead to unsafe movements or policy violations (Perez et al., 2022). They can include content filters, motion feasibility checks, emergency stops, or rule-based overrides to ensure the robot does not execute hazardous plans. The EU AI Act requires that high-risk AI systems incorporate appropriate human oversight to prevent or minimize risks (Article 14).

• Transparency/Explainability: The degree to which the internal decision-making of an LLM-based robot is understandable to humans. A transparent system provides human-interpretable reasons for its actions or recommendations–for example, showing the chain of thought or plan rationale in plain language (Department of Industry and Science, 2019). This often involves explainable AI techniques (e.g., natural language explanations or visualizations of what the robot “thought”) so that users and developers can trace why the robot behaved a certain way. Transparency is key for building user trust and enabling oversight, as people can follow the robot’s logic and catch potential errors or biases. The EU AI Act emphasizes transparency obligations, particularly for high-risk AI systems, requiring that their capabilities and limitations are communicated in a clear and accessible manner (Article 13).

• Auditability/Accountability: The capability to log, trace, and externally review the robot’s decisions and actions after the fact. An auditable LLM-based system maintains records or “audit trails” of its reasoning steps and behaviors (Strobel et al., 2024). This means that regulators or engineers can later inspect what the robot did and why, facilitating investigations of failures or undesirable outcomes. Auditability complements transparency by ensuring retrospective accountability–even if a robot acts autonomously in real time, its actions leave a verifiable trail that can be analyzed for compliance, bias, or error, thus enabling rigorous audits and improvements. Ultimately, an accountable system has clear provisions so that someone (or some process) can be held responsible for decisions and failures (Department of Industry and Science, 2019). The EU AI Act mandates that high-risk AI systems maintain comprehensive records to ensure traceability and facilitate post-market monitoring (Article 12).

Notably, the EU AI Act mandates many of these features for high-risk AI systems—such as robots operating in public spaces or healthcare—by requiring documented risk management, human-in-the-loop oversight, and transparency measures for decision-making. These same principles are reflected in the NIST AI Risk Management Framework^2^’s core functions (Identify, Govern, Detect, Respond, Recover), which prescribe continuous monitoring of AI performance, bias mitigation, and explainability across an AI system’s life cycle and in IEEE’s Ethically Aligned Design, which emphasizes accountability, human agency, and explicability of autonomous systems^3^. In our terms, the EU AI Act’s risk-management and oversight requirements align directly with our Safety Guardrails and Human Oversight metrics, while NIST’s Govern” function echoes our emphasis on fairness and auditability, and IEEE’s calls for explicability” map onto our Transparency and Explainability dimensions. Moreover, recent work in explainable neuro-robotics highlights concrete methods for tracing internal decision flows in embodied agents (Khan and Olds, 2023), and the field of Explainable Robotics has begun to formalize how robots should generate human-understandable rationales for their actions (Setchi et al., 2020). By situating our evaluation within these established governance frameworks and tapping into adjacent literature on embodied AI explainability, we demonstrate that our analysis is not merely academic: it anticipates—and in many respects meets—the real compliance requirements that regulators and policymakers are now putting into place.

### 4.2 Clustering of LLM-based robotic systems

Using the above metrics, we qualitatively assessed the LLM-based robotic systems from Tables 2–5 and grouped them into five clusters. Each cluster contains systems with similar levels of ethical safeguards or shortcomings. Table 6 below summarizes the clusters, listing representative papers and a qualitative rating (High/Moderate/Low) for each of the five metrics. These ratings are based on evidence in the papers (e.g., documented bias tests, explicit safety modules, explainability features) or, when not explicitly stated, our inference from the system’s design and stated capabilities.

**TABLE 6:** Clustering of LLM-Based Robotic Systems by Ethical, Safety, and Transparency Metrics. Low 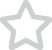, Moderate 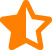, and High 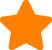 indicate the qualitative strength of each metric in the cluster.

Cluster name	Papers (Tables 2–5)	Fairness and bias	Safety guardrails	Transparency/Explainability	Auditability/Accountability
High Ethical Rigor and Safety Focus 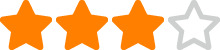	Manipulate-Anything, VADER, ${LLM}^{3}$ , VoxPoser	High	High	Moderate	Moderate
Governance and Compliance Oriented 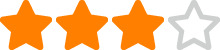	MALMM, RoCo, LaMI, LLM2Swarm	High	Moderate	Moderate	High
Transparency Interpretability Emphasis 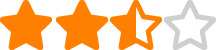	ProgPrompt, RONAR, Inner Monologue, LLM-MARS, RobotIQ	Moderate	Moderate	High	Moderate
Robust Safety Measures and Technical Alignment 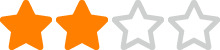	SayCan, RT-1, RT-2, RobotGPT, SayPlan, MOO	Moderate	High	Moderate	Low
Capability-Focused with Limited Ethical Measures 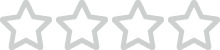	DrEureca, Code-as-Policies, LM-Nav, REAL, RoboCat, Gato, HULC++, Interactive Language, LLM-GROP, ChatGPT-for-Robotics, PALM-E	Low	Low	Low	Low

#### 4.2.1 High ethical rigor and safety focus

Systems in this cluster systematically integrate ethical evaluations and explicit safety protocols. They tend to build in bias mitigation and robust guardrails from the ground up. For instance, ${LLM}^{3}$ (Wang S. et al., 2024) incorporates safety triggers that automatically replan or halt execution when a generated plan might fail or go out of bounds. Similarly, Manipulate-Anything (Duan et al., 2024) performs multi-step manipulation with continuous self-verification–it actively checks the outcome of each action and retries or adjusts if an error is detected. VADER (Ahn et al., 2024) introduces a paradigm of robots seeking assistance: the robot uses visual checks to detect when it cannot safely proceed and then either asks a human or delegates to another robot, rather than risking an unsafe action. These projects all devote substantial effort to bias checks, fail-safes, and intervention mechanisms, achieving high fairness and safety by design. Their transparency and accountability tend to be moderate–for example, they may log failures for later analysis or provide basic explanations, but these features are less emphasized than proactive safety. The result is systems that prioritize aligned, safe behavior even if it means sacrificing some openness or convenience.

#### 4.2.2 Governance and compliance-oriented

This cluster emphasizes oversight and formal accountability, especially in complex or multi-robot scenarios. The included systems establish frameworks to monitor and enforce ethical behavior during operation. For example, MALMM (Singh et al., 2024) deploys three specialized LLM agents (Planner, Coder, Supervisor) that check and balance each other’s decisions, with a dedicated “Supervisor” agent overseeing executions and catching errors. RoCo (Mandi et al., 2024) and LLM2Swarm (Strobel et al., 2024) each maintain detailed audit trails of every agent action, providing traceability in case of failures or unintended behaviors. Systems such as LaMI (Wang C. et al., 2024) focus on cultural and social norm adherence–for instance, LaMI is explicitly designed to respect social etiquette and user preferences, achieving high fairness by customizing its responses to individual needs without bias. Meanwhile, ZeroCap (Venkatesh and Min, 2024) ensures compliance with external standards (e.g., it might enforce privacy or safety regulations in its domain). Across this cluster, governance mechanisms are paramount: they excel in providing human-in-the-loop checkpoints, rule-based interventions, or multi-agent consensus to keep the system’s behavior in check. Fairness is generally high (e.g., adhering to ethical norms or avoiding biased outcomes is often an objective), and these systems clearly assign responsibility for decisions. However, transparency and low-level safety may be only moderate–they do incorporate some explainability and basic robustness, but the priority is on structured oversight and meeting formal compliance criteria.

#### 4.2.3 Transparency and interpretability emphasis

Systems in this group are characterized by making the LLM’s decision process as understandable as possible to users, sometimes at the expense of other metrics. The goal in this cluster is to enhance user trust and facilitate auditing by revealing the robot’s reasoning. For example, ProgPrompt (Singh et al., 2023) plans robot tasks by producing human-readable pseudo-code or programs, so a human can literally read the generated plan to understand the intended steps. Likewise, LLM-MARS (Lykov et al., 2023) outputs an explicit behavior tree for multi-robot coordination, clearly delineating each agent’s role in the plan. RONAR Wang Z. et al. (2024) takes a different approach by narrating the robot’s actions in real time; as the robot acts, it provides a natural language explanation of what it is doing and why. These interpretability-focused techniques (structured plans, behavior trees, live narration) significantly enhance transparency–users or developers can follow along and spot errors or misunderstandings. That said, fairness and proactive safety are generally secondary in this cluster. The systems often do not implement advanced bias mitigation or safety-locking mechanisms, and some (like RONAR) do not even control actions directly, acting mainly as an explanatory layer. Thus, while they score high on transparency/interpretability, their safety and fairness measures tend to be moderate or limited. The implicit assumption is that by being more interpretable, issues can be noticed and corrected by humans, but the systems themselves currently rely on that human supervision for ethical assurance. Ongoing work is needed to integrate the high transparency with stronger built-in safeguards so that understandability does not come at the cost of reliability.

#### 4.2.4 Robust safety measures and technical alignment

This cluster contains systems that tackle safety and reliability as a technical alignment problem, tightly constraining the robot’s actions to what is known to be safe or feasible. They achieve high robustness through methods like skill grounding, strict constraints, or verification, but often pay less attention to bias or accountability. For instance, SayCan (Ahn et al., 2022) grounds the LLM’s high-level instructions in a library of verified skills and an affordance function–essentially, the LLM can only propose actions that a pretrained value model deems feasible and safe for the robot to execute. By filtering out impractical or risky commands, SayCan maintains a strong safety guarantee (the robot will not attempt something obviously dangerous or impossible). The MOO approach (Stone et al., 2023) further illustrates this technical alignment by explicitly grounding manipulation actions in vision-language embeddings, significantly enhancing robustness by constraining actions to objects identified clearly from language commands. Across this cluster, approaches like these yield high safety and reliability–the robots are very unlikely to do something catastrophically wrong, because the design limits them to vetted actions or heavily-trained policies. On the other hand, these systems typically offer only moderate transparency (the internal checks happen behind the scenes, though some may expose a reasoning trace) and minimal bias mitigation. Fairness is not a major focus because the tasks (e.g., object manipulation or navigation) do not inherently involve demographic decisions, and any potential biases in the LLM’s understanding are not explicitly audited. Governance or external accountability is also low–many of these works are technical proofs-of-concept that prioritize performance, so they often run autonomously without human oversight once deployed. In summary, this cluster’s philosophy is safety through design: by aligning LLM outputs with low-level controllers and environmental constraints, they ensure robust performance, but they do so in a way that is more implicit (inside the model) rather than through transparent ethics or oversight structures.

#### 4.2.5 Capability-focused with limited ethical measures

The final cluster comprises systems that prioritize raw capability and innovation in LLM-robot control, with minimal built-in ethical or transparency features. These works demonstrate impressive robotic performances or new general abilities, but they largely treat ethical and safety concerns as out-of-scope or leave them to future work. For instance, DrEureca (Ma et al., 2024) and Code-as-Policies (Liang et al., 2023) both focus on novel ways to improve robot learning (sim-to-real transfer in DrEureca’s case, and language-based policy generation in Code-as-Policies) and offer little discussion of bias, fairness, or explainability in their implementations. LM-Nav (Shah et al., 2023) and REAL (Tagliabue et al., 2024) advance long-horizon robot navigation and control via LLMs, but aside from using standard obstacle avoidance in the low-level controller, they do not incorporate special safety guardrails or ethical reasoning–the emphasis is on successfully reaching goals in open environments. Likewise, generalist agents like RoboCat (Bousmalis et al., 2023) and Gato (Reed et al., 2022) showcase multi-task learning across diverse domains, yet their design includes no specific mechanism for bias checking or transparency beyond the basic training data filtering. For instance, PaLM-E (Driess et al., 2023), despite its powerful multimodal reasoning capabilities, does not explicitly address ethical dimensions, relying instead on emergent behaviors derived from large-scale multimodal training. In this cluster, all four metrics are rated low. The systems typically run autonomously with no human oversight loop, have opaque reasoning (e.g., end-to-end neural policies or black-box prompts), and do not address potential unfair outcomes or unsafe instructions explicitly. It is worth noting that even in this cluster, authors often acknowledge the importance of ethics–for example, the team behind ChatGPT-for-Robotics (Vemprala et al., 2024) suggests that a human should review and approve the plans/code that ChatGPT generates before execution, and Interactive Language (Lynch et al., 2023) allows a human to iteratively correct the robot via dialogue. However, such measures are *ad hoc* or optional, not deeply embedded in the system’s architecture. Overall, the cluster reflects the reality that many cutting-edge LLM-robotics projects are still proof-of-concept level–excelling in capability demonstrations while only lightly touching on the ethical and safety implications.

#### 4.2.6 Summary of ethical, safety, and transparency across clusters

**FIGURE 2:**
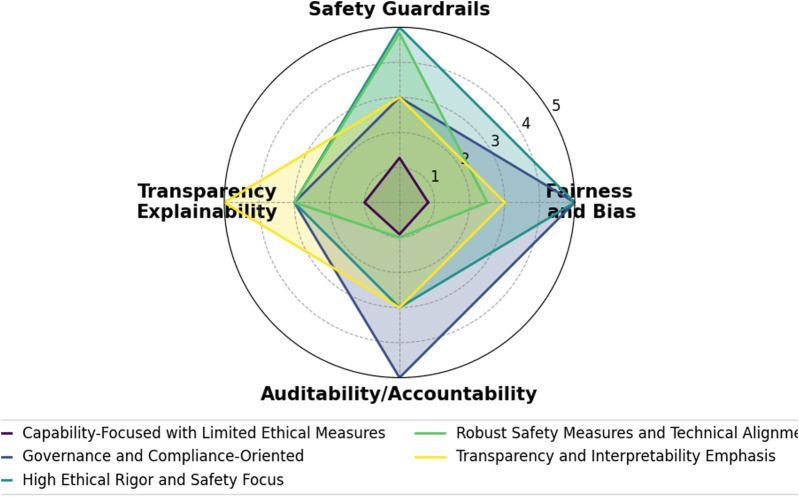
Radar plot summarizing the relative strengths of the five clusters of LLM-based robotic systems across four key ethical, safety, and transparency metrics.

Figure 2 provides a concise visual synthesis of the ethical, safety, and transparency attributes characterizing each of the five clusters previously discussed. This radar plot clearly highlights the key trade-offs present in current agentic LLM-based robotic research: the High Ethical Rigor and Safety Focus cluster distinctly leads in proactive Safety Guardrails and Fairness mitigation, whereas the Governance and Compliance-Oriented cluster excels predominantly in Auditability and Accountability measures. Conversely, clusters such as the Transparency and Interpretability Emphasis illustrate the critical role transparency plays in certain systems while still showing modest gaps in fairness and accountability. The Robust Safety Measures and Technical Alignment cluster demonstrates strong safety without correspondingly high transparency or accountability, reflecting a narrow technical alignment focus. Finally, the Capability-Focused with Limited Ethical Measures cluster presents the lowest scores across all dimensions, visually emphasizing the critical ethical gaps still prevalent in highly capable but minimally safeguarded robotic systems.

### 4.3 Ethical, safety, and transparency insights by task domain

**FIGURE 3:**
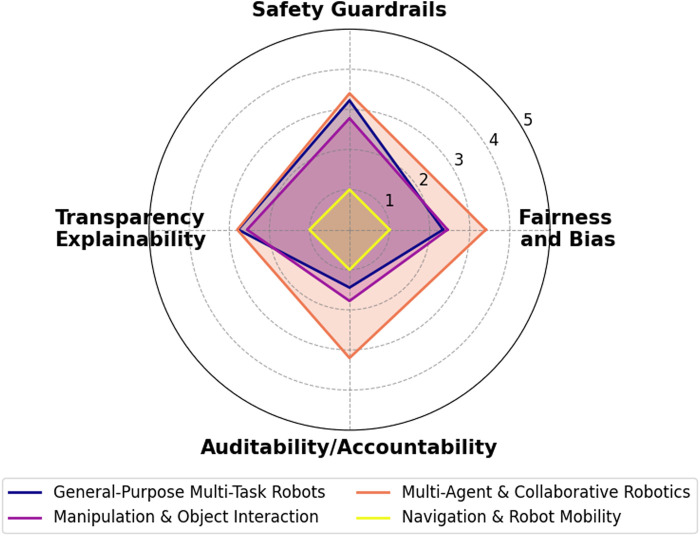
Radar plot summarizing ethical, safety, and transparency metrics across robotic task domains.

Figure 3 illustrates the comparative analysis of ethical, safety, and transparency considerations across the four key task domains. A notable insight is the clear variation in emphasis across these domains. The *Multi-Agent and Collaborative Robotics* domain demonstrates the highest overall ethical commitment, particularly excelling in Auditability/Accountability and Fairness due to built-in oversight mechanisms and explicit coordination roles. Conversely, *Navigation and Robot Mobility* consistently scored lowest across all dimensions, highlighting the significant gap between autonomy-focused applications and explicit ethical integration.

In terms of *Manipulation and Object Interaction* and *General-Purpose Multi-Task Robots*, these domains share moderate scores across most metrics, underscoring their focus on safety through technical robustness (particularly in manipulation) and adaptability via extensive model training (general-purpose), albeit with limited fairness, transparency, or governance considerations. The visualization emphasizes how ethical and transparency measures vary substantially according to the robotic task, clearly identifying targeted opportunities for advancing responsible AI practices within each distinct domain.

#### 4.3.1 Navigation and robot mobility

In *Navigation and Robot Mobility*, agentic LLM-based robots prioritize autonomy and reliable pathfinding, yet largely omit explicit ethical considerations. Systems typically integrate classical robotics methods for physical safety (e.g., obstacle avoidance), resulting in moderately robust performance. However, fairness is virtually neglected, mainly because demographic considerations are rarely acknowledged. Transparency and auditability are minimal, reflecting limited user insight into route selection and negligible mechanisms for human oversight or accountability.

Moving forward, there is a clear gap to address: navigation systems should integrate explanations of path selections and explicitly handle fairness concerns, particularly in socially sensitive navigation contexts (e.g., surveillance or assistive scenarios). Improved transparency would allow users to understand and trust navigation decisions, while auditability mechanisms (e.g., logging reasons for path choices) could bolster accountability significantly.

#### 4.3.2 Manipulation and object interaction

*Manipulation and Object Interaction* robots consistently emphasize technical safety and robustness, effectively integrating multimodal grounding or affordance-based filtering to limit unsafe actions. Techniques such as those employed by MOO and SayCan strongly illustrate this trend, resulting in comparatively high safety scores. However, the manipulation domain remains weak in fairness, transparency, and governance; few systems explicitly address demographic biases, and transparency usually depends on the specific approach (e.g., code-based outputs like ProgPrompt offer better interpretability).

Future research in manipulation robotics should explicitly address potential fairness issues emerging from ambiguous instructions and prioritize integrating explainability features into decision-making processes. Additionally, structured governance practices, like formal audits of robot actions and outcomes, could significantly enhance user trust and accountability.

#### 4.3.3 Multi-agent and collaborative robotics

*Multi-Agent and Collaborative Robotics* systems currently lead in ethical integration, particularly due to inherent coordination requirements which naturally lend themselves to auditability and fairness. This domain exhibits the highest governance scores through explicit supervisory roles, as exemplified by MALMM and RoCo, where agent interactions facilitate structured oversight. Transparency is inherently higher due to explicit agent communication in natural language or behavior trees, providing a clear trace of reasoning.

Despite these strengths, safety measures are often limited to systemic safeguards rather than explicit runtime safety constraints. Thus, opportunities remain to enhance explicit runtime safety guardrails and fairness considerations, particularly ensuring equitable task distributions and unbiased decision-making in team settings.

#### 4.3.4 General-purpose multi-task robots

*General-Purpose Multi-Task Robots* exhibit significant variability, with some systems prioritizing impressive adaptability through large-scale multimodal training (e.g., PaLM-E, RT-2) but exhibiting minimal explicit ethical safeguards or transparency. Such model-driven approaches frequently assume ethical alignment from broad training, an assumption posing substantial real-world deployment risks due to opaque decision-making and minimal governance.

Conversely, human-centric or modular frameworks like ChatGPT-for-Robotics incorporate clearer oversight and conversational transparency, although fairness remains largely unaddressed. This domain urgently requires scalable oversight strategies, more explicit fairness audits, and transparency methods that enable understanding of complex multimodal model behaviors, ensuring robust ethical alignment alongside expanding capabilities.

#### 4.3.5 Integrated observations and remarks

Overall, the domain-specific analysis, presented in Figure 3, reveals that ethics and safety considerations have not been uniformly addressed. For example, none of the navigation-focused papers explicitly tackled algorithmic fairness in path planning, which might be acceptable for simple tasks but could become a concern in social navigation (avoiding bias in whom a delivery robot prioritizes, for instance). In contrast, multi-agent robotics papers frequently discuss accountability mechanisms, since coordination failures can have safety repercussions in teams of robots or human-robot collaboration. Manipulation papers often lie in between, sometimes including safety stops (to avoid harmful actions), but rarely disclosing decision rationales. This variance suggests that ethical and transparency measures are driven more by the nature of the task than by any standard practice–highlighting a need for broader guidelines that transcend domains.

## 5 Open problems and emerging directions

**FIGURE 4:**
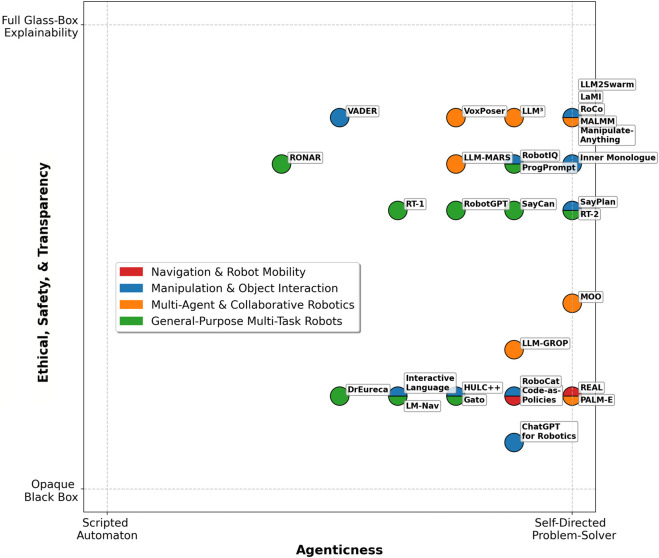
Competitiveness Graph of LLM-based robotic systems, showing their distribution across “Agenticness” (horizontal axis) and “Ethical, Safety, and Transparency” (vertical axis). Approaches are color-coded by their task domain highlighting the trade-offs between emergent autonomy and explainability.

Figure 4 maps a variety of LLM-based robotic systems along two axes: Agenticness (horizontal) and Ethical, Safety, and Transparency (vertical). The agenticness score was just retrieved from the first column of the tables 2 - 5, while for the Transparency, an average over all the metrics defined in subsection 4.1 is utilized.

At a glance, one notices four general clusters—corresponding roughly to the main task domains covered in this survey—with each approach occupying a distinct position depending on how “emergent” its autonomy is and how transparent or interpretable its decision-making becomes. Among our reviewed works, the ones that come closest to combining high agenticness and high transparency might be LLM2Swarm, LaMI, RoCo, MALMM, and Manipulate-Anything. Systems in *Multi-Agent and Collaborative Robotics* and *General-Purpose Multi-Task Robots* domains cluster toward high agentic capacity yet fall significantly short in transparency and ethical safeguards, whereas navigation and manipulation solutions tend to remain closer to the center, balancing autonomy with embedded safety checks.

### 5.1 Usual practices and trade-offs

Most existing pipelines still follow a design pattern where an LLM is used for high-level reasoning or planning, while traditional, rule-based modules handle low-level control and safety constraints. This hybrid approach has been effective in keeping systems safe (human or hard-coded control at lower levels) but inherently constrains the agentic potential of the LLM component. For example, typical “manipulation + LLM” configurations expose parts of their planning process (via affordance models or code-based outputs) but are constrained by pre-scripted motion primitives. Such a cautious strategy explains why these systems, as seen in the new graph, tend to lie toward the mid-range of both axes. Our analysis suggests a fundamental trade-off in current LLM-robotics architectures: boosting agentic autonomy often means introducing complexity and opacity that make the system harder to trust or verify. Conversely, insisting on transparency and strict oversight tends to limit the system’s autonomy. This trade-off is evident in the near absence of any system in the “high-high” quadrant of Figure 4. Bridging this gap is an open challenge. Possible research directions include: developing explainable LLMs (so that even autonomous decisions are interpretable) or designing layered control schemes where an agentic core is monitored by an explainable guardian system. So far, attempts like using code-generation (ProgPrompt) or modular policies (i.e., a symbolic planner supervising an LLM) have had mixed success–achieving some transparency at the cost of flexibility.

### 5.2 Void in the literature

A striking observation from Figure 4 is the near-emptiness of the upper-right quadrant—representing systems that achieve both high autonomy and robust transparency. In practice, highly agentic frameworks (especially in general-purpose and multi-agent settings) tend to depend on massive, opaque LLMs whose decision processes are not inherently interpretable. Conversely, systems that emphasize interpretability (such as code-based planners) often limit autonomy by confining the system to structured, semi-automatic behaviors. This gap underscores a crucial open problem: how to design frameworks that simultaneously deliver self-sufficient, high-level autonomy and built-in, real-time introspection or self-explanation capabilities. The graph clearly signals that without such integrated approaches, advanced robotic systems risk trading off one essential quality for the other.

Looking ahead, bridging this gap will require novel architectural innovations. Research should focus on embedding ethical, safety, and transparency measures as native design principles—not as *post hoc* add-ons via prompt engineering or symbolic traces. Emerging ideas such as self-explaining LLMs, real-time introspection loops, and open-source model audits appear promising in this respect. Furthermore, the domain-specific clustering revealed in the Figure 3 suggests that a one-size-fits-all approach is unlikely to succeed; tailored strategies that account for the inherent trade-offs in each task domain are needed. In particular, multi-agent and general-purpose systems must be rethought to mitigate the opacity of large-scale models while preserving their emergent capabilities.

In summary, the competitiveness graph of Figure 4 raises the following questions about the critical research gaps: How can we design future LLM-driven robotic systems that achieve a high degree of autonomous decision-making while remaining transparent and ethically robust? What methodologies can reconcile the inherent tension between emergent behavior and interpretability? We note that solving these issues will likely require multi-disciplinary efforts: advances in machine learning (to make LLMs more interpretable), in robotics (to ensure safety at the control level), and in governance (to establish standards and validation protocols for agentic AI). Early steps such as the EU AI Act’s requirements for transparency and human oversight are pushing developers in this direction, but the technical community must respond with innovations that make transparency intrinsic to highly autonomous systems, rather than an afterthought.

## 6 Conclusion

In summary, our survey reveals a field in transition: early successes in integrating LLMs into robotics hint at the immense potential for more autonomous, flexible machines, but they also expose significant gaps in ethical and transparent design. No current system fully satisfies the dual demand of high agentic autonomy and high accountability, meaning there is much work to do before we can trust these robots as true teammates. Key lessons include the importance of grounding LLMs in real-world feedback loops, the value of modular architectures for safety, and the urgent need for built-in explainability as autonomy increases. Standardised benchmarks that quantify prompt sensitivity and long-horizon consistency will be essential to translate agentic LLMs into safety-critical deployments.

Moving forward, we advocate for research that addresses these challenges head-on. This includes creating better evaluation metrics that cover both performance and ethical criteria, designing new algorithms for real-time robot self-explanation, and conducting longitudinal real-world trials to study safety in practice. Achieving the right balance will indeed require multi-disciplinary collaboration–engineers, ethicists, and regulators must work in concert. Encouragingly, the very capabilities that make LLM-driven robots powerful (language understanding and reasoning) could be harnessed to encode human norms and oversight mechanisms into their operation. If guided by the insights and recommendations outlined in this survey, the next-generation of agentic AI robots could be not only more capable than ever but also demonstrably safe, fair, and transparent, earning the trust required for widespread deployment.

## Footnotes

1
https://eur-lex.europa.eu/eli/reg/2024/1689/oj

2
https://www.nist.gov/itl/ai-risk-management-framework

3
https://standards.ieee.org/industry-connections/activities/ieee-global-initiative/

## References

[B1] Acharya D. B. Kuppan K. Divya B. (2025). Agentic ai: autonomous intelligence for complex goals–a comprehensive survey. IEEE Access 13, 18912–18936. 10.1109/access.2025.3532853

[B2] Ahn M. Arenas M. G. Bennice M. Brown N. Chan C. David B. (2024). Vader: visual affordance detection and error recovery for multi robot human collaboration. arXiv Prepr. arXiv:2405.16021.

[B3] Ahn M. Brohan A. Brown N. Chebotar Y. Cortes O. David B. (2022). Do as i can, not as i say: grounding language in robotic affordances. arXiv Prepr. arXiv:2204.01691.

[B4] Azeem R. Hundt A. Mansouri M. Brandão M. (2024). Llm-driven robots risk enacting discrimination, violence, and unlawful actions. arXiv Prepr. arXiv:2406.08824.

[B5] Bousetouane F. (2025). Agentic systems: a guide to transforming industries with vertical ai agents. arXiv Prepr. arXiv:2501.00881.

[B6] Bousmalis K. Vezzani G. Rao D. Devin C. Lee A. X. Bauzá M. (2023). Robocat: a self-improving generalist agent for robotic manipulation. arXiv Prepr. arXiv:2306.11706.

[B7] Brohan A. Brown N. Carbajal J. Chebotar Y. Chen X. Choromanski K. (2023). Rt-2: vision-language-action models transfer web knowledge to robotic control. arXiv Prepr. arXiv:2307.15818.

[B8] Brohan A. Brown N. Carbajal J. Chebotar Y. Dabis J. Finn C. (2022). Rt-1: robotics transformer for real-world control at scale. arXiv Prepr. arXiv:2212.06817.

[B9] Cheng G. Zhang C. Cai W. Zhao L. Sun C. Bian J. (2024). Empowering large language models on robotic manipulation with affordance prompting. arXiv Prepr. arXiv:2404.11027.

[B10] Department of Industry I. Science A. G. (2019). Accountability: ai ethics principles

[B11] Ding Y. Zhang X. Paxton C. Zhang S. (2023). “Task and motion planning with large language models for object rearrangement,” in 2023 IEEE/RSJ international conference on intelligent robots and systems (IROS) (IEEE), 2086–2092.

[B12] Dong Y. Mu R. Zhang Y. Sun S. Zhang T. Wu C. (2024). Safeguarding large language models: a survey. arXiv Prepr. arXiv:2406.02622. 10.1007/s10462-025-11389-2 PMC12532640 41114380

[B13] Driess D. Xia F. Sajjadi M. S. Lynch C. Chowdhery A. Wahid A. (2023). Palm-e: an embodied multimodal language model

[B14] Duan J. Yuan W. Pumacay W. Wang Y. R. Ehsani K. Fox D. (2024). Manipulate-anything: automating real-world robots using vision-language models. arXiv Prepr. arXiv:2406.18915.

[B15] Haslum P. Lipovetzky N. Magazzeni D. Muise C. Brachman R. Rossi F. (2019). An introduction to the planning domain definition language. arXiv 13.

[B16] Hu Y. Xie Q. Jain V. Francis J. Patrikar J. Keetha N. (2023). Toward general-purpose robots via foundation models: a survey and meta-analysis. arXiv Prepr. arXiv:2312.08782.

[B17] Huang Q. Wake N. Sarkar B. Durante Z. Gong R. Taori R. (2024). Position paper: agent ai towards a holistic intelligence. arXiv Prepr. arXiv:2403.00833.

[B18] Huang W. Wang C. Zhang R. Li Y. Wu J. Fei-Fei L. (2023). Voxposer: composable 3d value maps for robotic manipulation with language models. arXiv Prepr. arXiv:2307.05973.

[B19] Huang W. Xia F. Xiao T. Chan H. Liang J. Florence P. (2022a). Inner monologue: embodied reasoning through planning with language models. arXiv Prepr. arXiv:2207.05608.

[B21] Hundt A. Agnew W. Zeng V. Kacianka S. Gombolay M. (2022). “Robots enact malignant stereotypes,” in Proceedings of the 2022 ACM conference on fairness, accountability, and transparency, 743–756.

[B22] Jeong H. Lee H. Kim C. Shin S. (2024). A survey of robot intelligence with large language models. Appl. Sci. 14, 8868. 10.3390/app14198868

[B23] Jin Y. Li D. Yong A. Shi J. Hao P. Sun F. (2024). Robotgpt: robot manipulation learning from chatgpt. IEEE Robotics Automation Lett. 9, 2543–2550. 10.1109/lra.2024.3357432

[B24] Khan M. S. Olds J. L. (2023). When neuro-robots go wrong: a review. Front. Neurorobotics 17, 1112839. 10.3389/fnbot.2023.1112839 PMC9935594 36819005

[B25] Kim S. S. Liao Q. V. Vorvoreanu M. Ballard S. Vaughan J. W. (2024a). ““i’m not sure, but…”: examining the impact of large language models’ uncertainty expression on user reliance and trust,” in Proceedings of the 2024 ACM conference on fairness, accountability, and transparency, 822–835.

[B26] Kim Y. Kim D. Choi J. Park J. Oh N. Park D. (2024b). A survey on integration of large language models with intelligent robots. Intell. Serv. Robot. 17, 1091–1107. 10.1007/s11370-024-00550-5

[B27] Li P. An Z. Abrar S. Zhou L. (2025). Large language models for multi-robot systems: a survey. arXiv Prepr. arXiv:2502.03814.

[B28] Liang J. Huang W. Xia F. Xu P. Hausman K. Ichter B. (2023). “Code as policies: language model programs for embodied control,” in 2023 IEEE international conference on robotics and automation (ICRA) (IEEE), 9493–9500.

[B29] Lykov A. Dronova M. Naglov N. Litvinov M. Satsevich S. Bazhenov A. (2023). Llm-mars: large language model for behavior tree generation and nlp-enhanced dialogue in multi-agent robot systems. arXiv Prepr. arXiv:2312.09348.

[B30] Lynch C. Wahid A. Tompson J. Ding T. Betker J. Baruch R. (2023). Interactive language: talking to robots in real time. IEEE Robotics Automation Lett., 1–8. 10.1109/lra.2023.3295255

[B31] Ma Y. J. Liang W. Wang H.-J. Wang S. Zhu Y. Fan L. (2024). Dreureka: language model guided sim-to-real transfer. arXiv Prepr. arXiv:2406.01967.

[B32] Mandi Z. Jain S. Song S. (2024). “Roco: dialectic multi-robot collaboration with large language models,” in 2024 IEEE international conference on robotics and automation (ICRA) (IEEE), 286–299.

[B33] Mees O. Borja-Diaz J. Burgard W. (2023). “Grounding language with visual affordances over unstructured data,” in 2023 IEEE international conference on robotics and automation (ICRA) (IEEE), 11576–11582.

[B34] Perez E. Huang S. Song F. Cai T. Ring R. Aslanides J. (2022). Red teaming language models with language models. arXiv Prepr. arXiv:2202.03286.

[B35] Rana K. Haviland J. Garg S. Abou-Chakra J. Reid I. Suenderhauf N. (2023). Sayplan: grounding large language models using 3d scene graphs for scalable robot task planning. arXiv Prepr. arXiv:2307.06135.

[B36] Raptis E. K. Kapoutsis A. C. Kosmatopoulos E. B. (2025). Robotiq: empowering mobile robots with human-level planning for real-world execution. arXiv Prepr. arXiv:2502.12862.

[B37] Reed S. Zolna K. Parisotto E. Colmenarejo S. G. Novikov A. Barth-Maron G. (2022). A generalist agent. arXiv Prepr. arXiv:2205.06175.

[B38] Ren A. Z. Dixit A. Bodrova A. Singh S. Tu S. Brown N. (2023). Robots that ask for help: uncertainty alignment for large language model planners. arXiv Prepr. arXiv:2307.01928.

[B39] Setchi R. Dehkordi M. B. Khan J. S. (2020). Explainable robotics in human-robot interactions. Procedia Comput. Sci. 176, 3057–3066. 10.1016/j.procs.2020.09.198

[B40] Shah D. Osiński B. Levine S. (2023). “Lm-nav: robotic navigation with large pre-trained models of language, vision, and action,” in Conference on robot learning (PMLR), 492–504.

[B41] Shavit Y. Agarwal S. Brundage M. Adler S. O’Keefe C. Campbell R. (2023). “Practices for governing agentic ai systems,”. OpenAI. Research Paper.

[B42] Singh H. Das R. J. Han M. Nakov P. Laptev I. (2024). Malmm: multi-agent large language models for zero-shot robotics manipulation. arXiv Prepr. arXiv:2411.17636.

[B43] Singh I. Blukis V. Mousavian A. Goyal A. Xu D. Tremblay J. (2023). “Progprompt: generating situated robot task plans using large language models,” in 2023 IEEE international conference on robotics and automation (ICRA) (IEEE), 11523–11530.

[B44] Stone A. Xiao T. Lu Y. Gopalakrishnan K. Lee K.-H. Vuong Q. (2023). Open-world object manipulation using pre-trained vision-language models. arXiv Prepr. arXiv:2303.00905.

[B45] Strobel V. Dorigo M. Fritz M. (2024). Llm2swarm: robot swarms that responsively reason, plan, and collaborate through llms. arXiv Prepr. arXiv:2410.11387.

[B46] Tagliabue A. Kondo K. Zhao T. Peterson M. Tewari C. T. How J. P. (2024). “Real: resilience and adaptation using large language models on autonomous aerial robots,” in 2024 IEEE 63rd conference on decision and control (CDC) (IEEE), 1539–1546.

[B47] Vemprala S. H. Bonatti R. Bucker A. Kapoor A. (2024). Chatgpt for robotics: design principles and model abilities. Ieee Access 12, 55682–55696. 10.1109/access.2024.3387941

[B48] Venkatesh V. L. Min B.-C. (2024). Zerocap: zero-shot multi-robot context aware pattern formation via large language models. arXiv Prepr. arXiv:2404.02318.

[B49] Wang C. Hasler S. Tanneberg D. Ocker F. Joublin F. Ceravola A. (2024a). “Lami: large language models for multi-modal human-robot interaction,” in Extended abstracts of the CHI conference on human factors in computing systems, 1–10.

[B50] Wang S. Han M. Jiao Z. Zhang Z. Wu Y. N. Zhu S.-C. (2024b). “LlmË† 3: large language model-based task and motion planning with motion failure reasoning,” in 2024 IEEE/RSJ international conference on intelligent robots and systems (IROS) (IEEE), 12086–12092.

[B51] Wang Z. Liang B. Dhat V. Brumbaugh Z. Walker N. Krishna R. (2024c). I can tell what i am doing: toward real-world natural language grounding of robot experiences. arXiv Prepr. arXiv:2411.12960.

[B52] Zeng F. Gan W. Wang Y. Liu N. Yu P. S. (2023). Large language models for robotics: a survey. arXiv Prepr. arXiv:2311.07226.

